# Deep learning techniques and mathematical modeling allow 3D analysis of mitotic spindle dynamics

**DOI:** 10.1083/jcb.202111094

**Published:** 2023-03-02

**Authors:** David Dang, Christoforos Efstathiou, Dijue Sun, Haoran Yue, Nishanth R. Sastry, Viji M. Draviam

**Affiliations:** 1 School of Biological and Behavioural Sciences, Queen Mary University of London, London, UK; 2 Department of Informatics, King’s College London, London, UK

## Abstract

Time-lapse microscopy movies have transformed the study of subcellular dynamics. However, manual analysis of movies can introduce bias and variability, obscuring important insights. While automation can overcome such limitations, spatial and temporal discontinuities in time-lapse movies render methods such as 3D object segmentation and tracking difficult. Here, we present SpinX, a framework for reconstructing gaps between successive image frames by combining deep learning and mathematical object modeling. By incorporating expert feedback through selective annotations, SpinX identifies subcellular structures, despite confounding neighbor-cell information, non-uniform illumination, and variable fluorophore marker intensities. The automation and continuity introduced here allows the precise 3D tracking and analysis of spindle movements with respect to the cell cortex for the first time. We demonstrate the utility of SpinX using distinct spindle markers, cell lines, microscopes, and drug treatments. In summary, SpinX provides an exciting opportunity to study spindle dynamics in a sophisticated way, creating a framework for step changes in studies using time-lapse microscopy.

## Teaser

Spatial and temporal discontinuities in time-lapse movies frequently disrupt automation methods such as 3D object segmentation and object tracking. To overcome this hurdle, we introduced SpinX, an image analysis framework to combine deep learning and mathematical object modeling to track mitotic spindle movements in 3D.

## Introduction

Computational image analysis tools and single-cell imaging methods can accelerate cell biology studies (Carpenter et al., 2006; Held et al., 2010; Ren et al., 2021 *Preprint*) and drug discovery efforts (Caicedo et al., 2017). Although deep learning (DL) has already revolutionized the automated analysis of still microscopy images for high-throughput object identification (Ronneberger et al., 2015; Schmidt et al., 2018; Stringer et al., 2021; Yang et al., 2020), this advance is only beginning to be extended to time-lapse microscopy movies for analyzing structural dynamics of objects through time and 3D space (Lefebvre et al., 2021). Extending DL approaches to time-lapse movies has faced at least two critical hurdles: first, the precise continuous tracking of structures through time requires tailored 3D object modeling tools to overcome spatial and temporal discontinuities that are intrinsic to time-lapse 3D movies of fast-moving objects. Second, feature-rich analysis supported by DL methods requires large volumes of high-resolution time-lapse movie datasets (Goswami et al., 2017). Nevertheless, as DL architectures for still images of fixed-cells (LeCun et al., 2015; Moen et al., 2019; von Chamier et al., 2021) have helped overcome the drawback of manual analysis (with respect to image segmentation which is inherently tedious, slow, and error-prone), developing new DL architectures for time-lapse movies of live-cells can advance quantitative 3D studies of subcellular and cellular dynamics.

Automated tools to analyze dynamic changes in intensities captured in live-cell movies are available (Cai et al., 2018; Held et al., 2010; Walther and Ellenberg 2018). However, tools that can reliably track precise changes in 3D shape and motion of objects within dividing cells are challenging to develop. Particularly, in specimens where phototoxicity or photobleaching limits the frequent acquisition of 3D images (Icha et al., 2017), spatiotemporal sampling is severely restricted. To overcome this limitation, spatially and temporally discontinuous time-lapse movies with limited axial sampling are preferred. For instance, fluorescent labeling of dividing cells with condensed chromosomes or long-term high-resolution imaging of proliferating cells is well known to induce phototoxicity (Hart et al., 2021; Progatzky et al., 2013). Consequently, dividing cells are not continuously imaged in high-resolution as full volume data, which results in missing data that disallows 3D tracking of subcellular movements, subsequently impairing our full understanding of mitotic defects or the development of anti-mitotic drugs (Iorio et al., 2015; Patel et al., 2016; Tamura et al., 2015).

The mitotic spindle is a complex and dynamic structure that is dependent on the function and regulation of multiple factors: the microtubule cytoskeleton (Tamura and Draviam, 2012), molecular motors (Fielmich et al., 2018; Laan et al., 2012; Okumura et al., 2018), actin clouds (Kwon et al., 2015), cell cortex rigidity (Kunda et al., 2008), cell–cell adhesion complexes (Théry et al. 2007; Théry et al., 2005), and chromosome congression (McEwen et al., 2001). The mitotic spindle undergoes complex 3D movements in longitudinal, equatorial, and axial directions, by integrating both intracellular and extracellular cues (Corrigan et al., 2013; Dimitracopoulos et al., 2020; Kiyomitsu and Cheeseman 2012; Kotak et al., 2012, Zulkipli et al., 2018) that ultimately guide the spindle to a final position which defines the plane of cell division (Chin et al., 2014; di Pietro et al., 2016). Being able to track and measure spindle movements can help us uncover the molecular cues that guide and power spindle rotation and centering movements in mammalian cells (Zulkipli et al., 2018). In addition, the complex 3D movements of the mitotic spindle make it an ideal subcellular model for testing the efficacy of DL-based video analysis methods aimed at extracting reliable and dynamic 3D information. Mammalian spindle volume is a good indicator of chromatin and cell volume (Kletter et al., 2022), and therefore a spindle tracker tool can generate a wide impact in cell biology studies across multiple cell types.

As DL methods are data hungry (Adadi, 2021), we first generated a large dataset of high-resolution time-lapse movies of mitotic spindle movements in human epithelial cells expressing a fluorescently tagged microtubule marker protein, Tubulin. Using this large dataset of 28,350 images, we built a comprehensive and extensible computational framework, SpinX, which bridges the gaps between discontinuous frames in time-lapse movies by utilizing state-of-the-art DL technologies and mathematical object modeling for 3D reconstruction of the mitotic spindle and cell cortex. Through stepwise benchmarking and detailed manual assessments, we demonstrated the potential of the 3D reconstruction module in overcoming spatiotemporal discontinuity in time-lapse movies of mitotic spindle and cell cortex. We established the generalization capacity of the SpinX framework for spindle segmentation using different microtubule-associated molecular markers, cell types, and microscopy systems. Finally, using SpinX to track 3D movements of the spindle in cells treated with CENP-E kinesin or MARK2 kinase inhibitor, we highlighted the strengths of AI-based time-lapse movie analysis in accelerating cytoskeletal research and drug development.

## Results

### Computational framework to track 3D movements of the mitotic spindle

**Figure 1.**
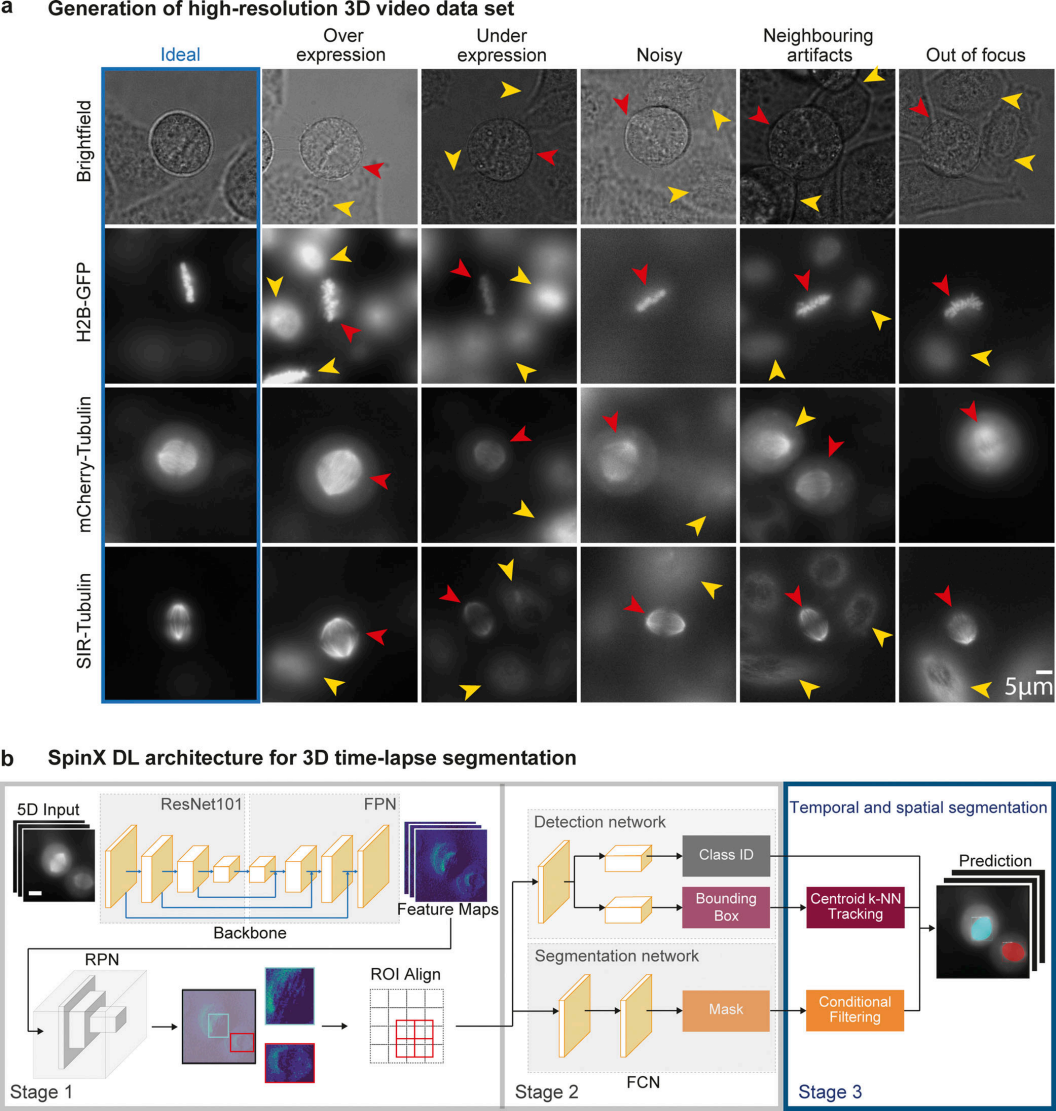
**Video dataset and DL architecture for spindle and cell cortex image segmentation. (a)** Representative images show complex variations in illumination and marker intensities intrinsic to time-lapse movies of subcellular structures (cell cortex, brightfield [label-free]; chromosomes, H2B-GFP; mitotic spindle, mCherry-Tubulin, or SiR-Tubulin dye). Ideal images are shown within the blue box. Red and yellow arrowheads indicate the object of interest and interfering variation(s), respectively. Scale bar: 5 μm. **(b)** DL model architecture of SpinX. The model expands the pre-existing Mask R-CNN architecture (ResNet101, FPN, RPN, ROI Align, and FCN [He et al., 2018]), by introducing a third stage (blue box). In Stage 1, the network performs object detection followed by the segmentation of spindle and cell cortex in Stage 2. Stage 3 (highlighted in blue) links temporal and spatial information in 3D live-cell movies through tracking and generates a consistent mask of the same object through time. The inputs of the model are grayscale or RGB images of various sizes (5D input). The outputs are binary masks of the same size as inputs with predicted foreground regions, bounding box coordinates (rectangular boxes in teal and red, Stage 1) and the corresponding Class ID (Stage 2). Scale bar: 10 µm.

Conventional computational methods (Driscoll and Danuser, 2015; Kervrann et al., 2016; Meijering et al., 2016, Youssef et al., 2011) have not been successful in continuous automated tracking of the mitotic spindle largely due to the lack of spatiotemporal continuity of 3D objects in time-lapse movies. To overcome spatial discontinuities in 3D images, spindle tracking tools have relied on manually ascertaining spindle poles (Corrigan et al., 2013; Jüschke et al., 2014) or have been limited to 2D tracking (Larson and Bement 2017), where DL approaches have not been used so far (Table S1). To create a computational framework for accurately tracking spindle movements in 3D, we first generated our own training dataset of high-resolution time-lapse movies for building the DL network. For this purpose, we labeled a Histone-2B-GFP (a chromosome marker) expressing HeLa cervical epithelial human cell line (Corrigan et al., 2013) with one of two different markers for the mitotic spindle, mCherry-Tubulin or SiR-Tubulin dye. Both markers have been established to decorate the microtubules of the mitotic spindle but with varying intensities (Corrigan et al., 2013; Stiff et al., 2020). The cell cortex was tracked label-free using brightfield images. A total of nearly five Terabytes of time-lapse movies were generated by imaging spindles in hundreds of cells exposed to MG132 (a proteasome inhibitor to prevent metaphase–anaphase transition [Hagting et al., 2002]). To closely reproduce challenges observed in large-scale high-throughput imaging screens, we built relatively long-term high-resolution time-lapse movies with three *z*-slice images (a *z*-gap of 2 μm). Image stacks were acquired once every 3 min to ensure that no obvious phototoxicity or photobleaching was introduced, and also that the discontinuity in time-lapse movies did not impair manual tracking of spindle pole movements. As expected, although live-cell movies are powerful in revealing dynamic cellular behavior, they capture highly heterogeneous information across and within cells through time (Fig. 1 a), making it difficult to quantitatively track spindle movements in 3D using traditional image segmentation methods (Corrigan et al., 2013). We observed several challenges in segmenting live-cell imaging data using traditional image analysis tools: (i) variability in sample illumination and protein expression between cells, where occasionally signal intensity can be highly non-uniform; (ii) noise from neighboring objects exacerbating low signal-to-noise ratios; and (iii) loss of focus resulting in blurry images due to natural 3D movements over time (Fig. 1 a). To overcome these challenges in tracking subcellular dynamics in high-throughput large-scale screens, we developed SpinX’s AI module by adapting the state-of-the-art Mask R-CNN DL architecture (He et al., 2018; see below and Fig. 1 b).

SpinX’s AI architecture identifies fluorescently-labeled spindles within label-free single-cell compartments by integrating three stages (Fig. 1 b), where the first two stages closely resemble a “native” Mask R-CNN-based DL architecture (for details see Materials and methods). The first stage combines a convolutional backbone architecture—comprised of a Residual Network with 101 layers (ResNet101; He et al., 2016) and a Feature Pyramid Network (FPN; Lin et al., 2017) with a Region Proposal Network (RPN; Ren et al., 2017). The aligned regions of interest (ROIs) are then passed onto the second stage of SpinX’s architecture: a Fully Convolutional Network (FCN; Shelhamer et al., 2017) that simultaneously performs object classification and segmentation for every aligned ROI (Fig. 1 b). The classes, bounding box information, and masks generated through the first two stages of SpinX’s DL architecture are passed to the third and final stage consisting of two modules (refer to blue box Fig. 1 b). In Stage 3, a “Conditional Filtering” module filters and discards detected objects based on their (i) confidence score that is derived from the prediction of the network; (ii) associated pixel count (i.e., area) that eliminates any artifacts including objects much smaller than the spindle; and (iii) location within the image canvas that allows the elimination of any detected object close to the image border as it would exhibit an incomplete shape. A second module in Stage 3, “Centroid *k*-NN Tracking” exploits the bounding box information, wherein the predicted bounding box centroid coordinates are fed into a single point k-nearest neighbors algorithm (*k*-NN) for tracking a singular detected object through time.

**Figure S1.**
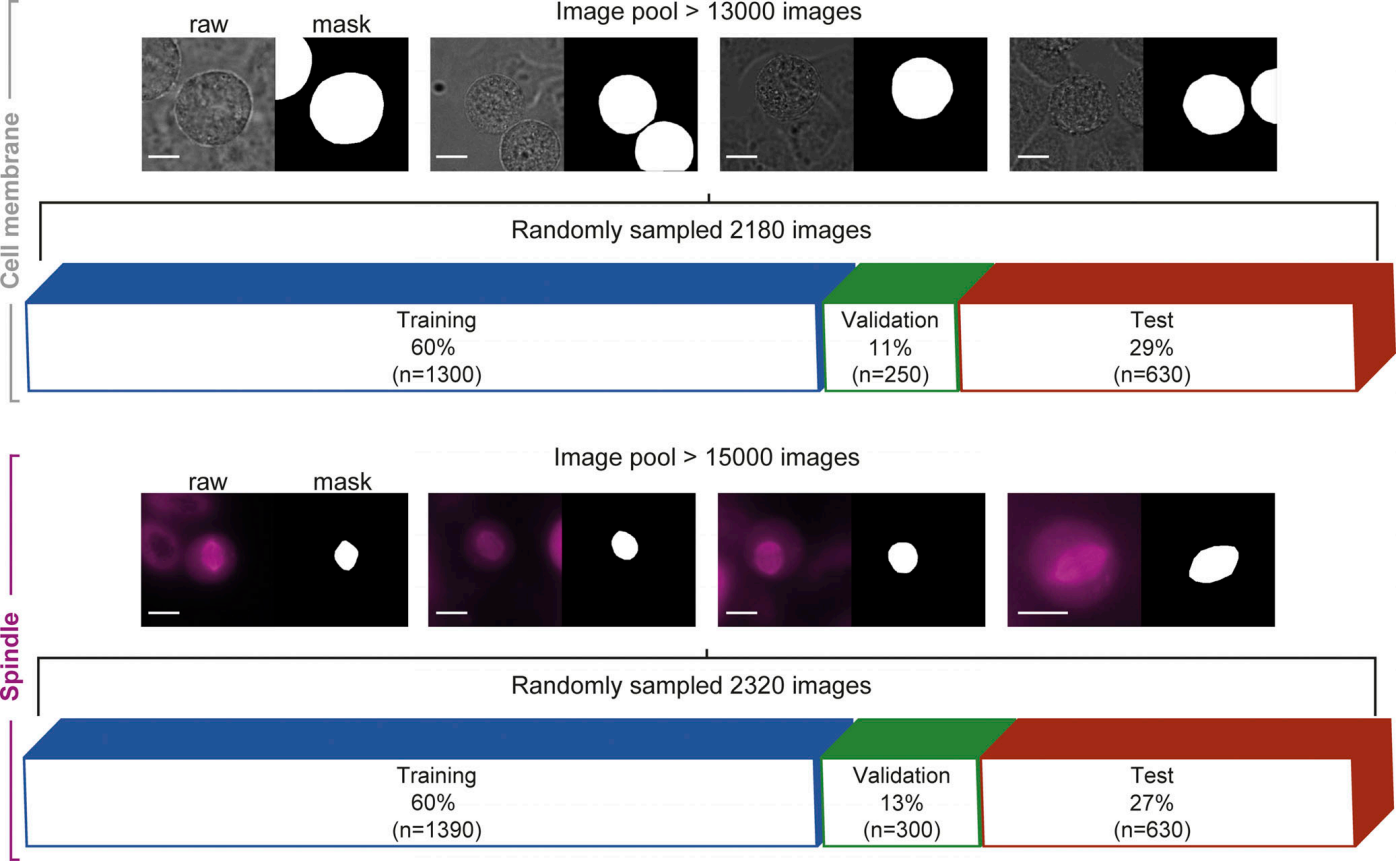
**Dataset composition.** Dataset composition of cell membrane (top row) and spindle (bottom row) images with their corresponding masks for training, validation, and testing. *n* corresponds to the number of images that were randomly selected from the image pools. Scale bars: 10 µm.

**Figure S2.**
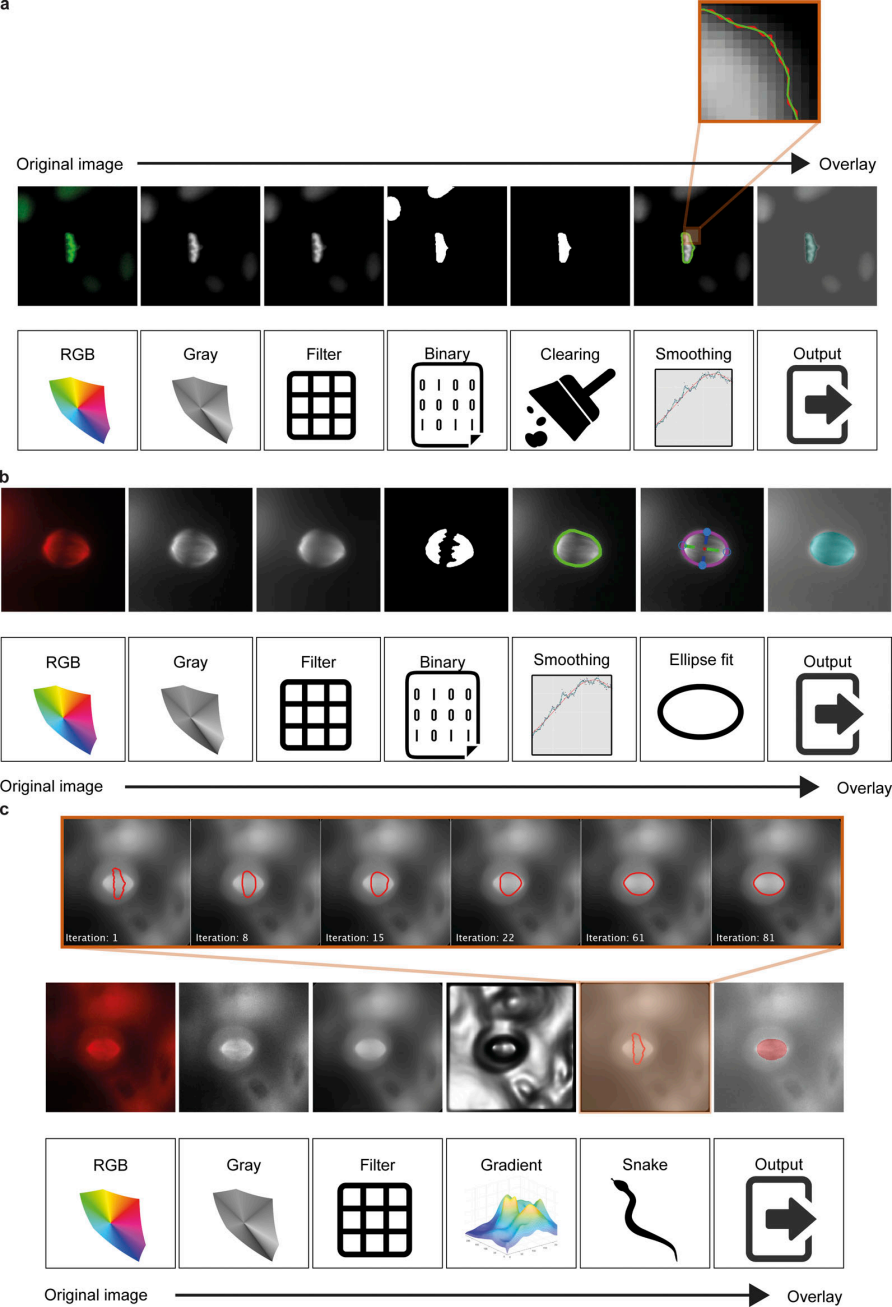
**SpinX pipelines for automated label generation. (a)** Conventional image processing pipeline to segment chromosomes. Pipeline includes using a median filter to reduce surrounding noise while preserving information of edges; performing Otsu’s thresholding to create a binary image; removing incomplete objects located at the image boundary; extracting boundary pixel information of the metaphase plate and applying a Savitzky-Golay filter to smoothen the boundary. **(b)** Conventional image processing pipeline to segment SiR-Tubulin-labeled spindle images. Pipeline includes using a median filter to reduce surrounding noise while preserving information of edges; performing Otsu’s thresholding to create a binary image; calculating the binary convex hull image; extracting boundary pixel information of the spindle; applying a Savitzky-Golay signal processing filter for smoothing; and utilizing an ellipse fit to obtain the final boundary information. **(c)** Conventional image processing pipeline (non-AI-based image processing techniques) to segment the mCherry-Tubulin-labeled spindle images. Pipeline includes applying a Gaussian filter to reduce surrounding noise while preserving information of edges; calculating the image gradient; using the segmentation mask of the metaphase plate to initiate the inverse snake; extracting boundary pixel information of the spindle and utilizing an ellipse fit to obtain the final boundary information. Scale bars: 10 µm.

**Figure S3.**
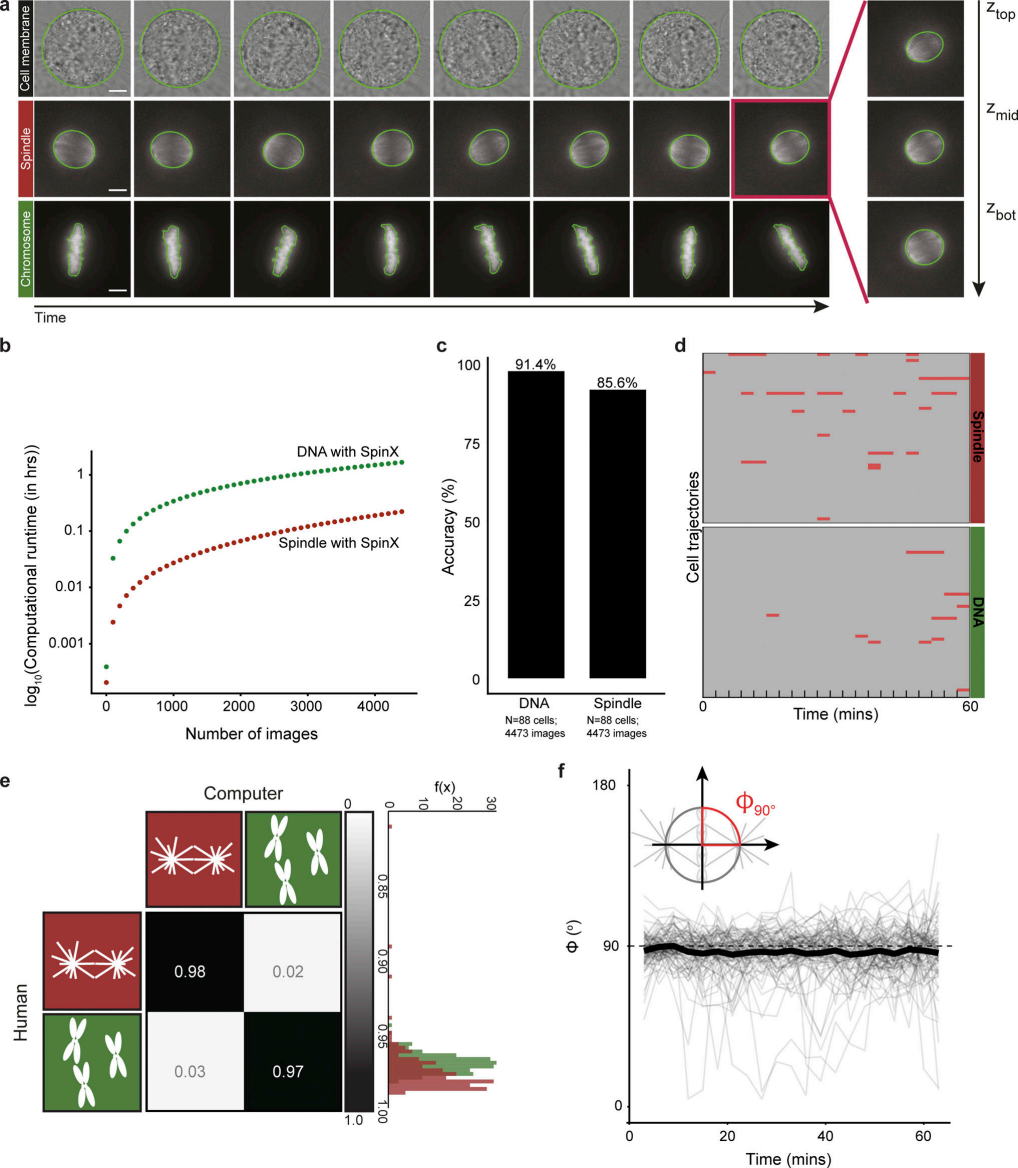
**Evaluation of automated label generation with SpinX. (a)** Time-lapse image stills of cell membrane, spindle (SiR-Tubulin), and chromosomes. The segmentation results are outlined in green. **(b)** Graph shows computational run time for chromosome (green) and spindle (red) annotation generation using SpinX. Note a log scale has been applied on the y-axes. Each dot indicates an additional 100 images. **(c)** Bar graph shows image-wise accuracy for chromosome and spindle channels. An image is defined as mis-segmented if SpinX fails to segment the full object’s boundaries or if the properties (e.g., orientation) are incorrect. Accuracy percentages are calculated by the number of mis-segmented frames over the total number of frames. **(d)** Automated chromosome and spindle segmentation of cell trajectories over time without correction. Mis-segmented frames are highlighted in red. The time interval between each frame is 3 min. **(e)** The correlation matrix displays the calculated correlation coefficient *ρ* which denotes the matching between human segmentation versus automated segmentation. The histogram shows the distribution of *ρ* grouped by channels. **(f)** Time series graph shows chromosome-spindle formation at metaphase. Dash-line at angle *φ* = 90° represents perfect perpendicular properties between spindle and chromosomes. *N* = 4,473 images per channel. Data obtained from 88 movies of metaphase cells over at least six independent experiments.

SpinX’s workflow is comprehensive, including pre-processing of data with annotations to 3D modeling. For training, validation, and testing of the pipeline, we used a total of 2,180 label-free (brightfield) images to deduce the cell membrane (randomly selected from an image pool of 13,230 images) and 2,320 fluorescently- or dye-labeled microtubules images to deduce the mitotic spindle (randomly selected from an image pool of 15,120 images; Fig. S1). Annotation of our training dataset (*n* = 1,300 images for cell cortex model; *n* = 1,390 images for spindle model) was carried out automatically and subsequently corrected manually (refer “Annotations” in Materials and methods). For automated label generation, we combined conventional image processing methods for specifically annotating chromosome and dye-based spindle (SiR-Tubulin) images (Fig. S2). The rate of automated labeling was nearly 100-fold faster than manual labeling that consumed 40–50 s for every image (Fig. S3, a and b). Our automated label generation pipeline correctly labeled chromosomes and dye-based spindle images with an accuracy of 91.4% and 85.6%, respectively (Fig. S3 c). All labels were manually assessed and subsequently corrected by experts (Fig. S3 d). Finally, using two orthogonal methods, we conducted performance measurements for object classification. First, using a correlation matrix to compare automated versus human segmentation outcomes, we found a high correlation coefficient denoting a strong match for both spindle and chromosome categories (Fig. S3 e). Second, using a priori information of the orientation of the spindle pole-to-pole axis and metaphase chromosome plate axis, we confirmed the extent of perpendicular properties between the two automatically annotated objects (mitotic spindle and chromosome plate) in 88 time-lapse movies from at least six independent repeats (Fig. S3 f). In summary, the manual and computational evaluation efforts together demonstrate a high accuracy with which automated labels are generated using SpinX.

### Benchmarking and refining annotation of classes improved SpinX AI performance

**Figure S4.**
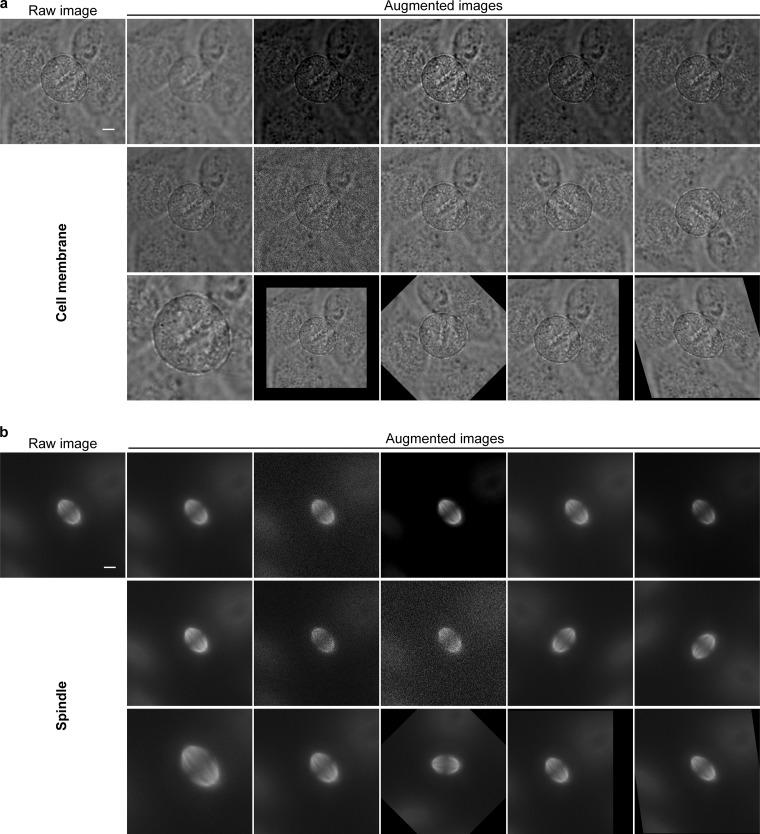
**Data augmentation. (a)** The data augmentation techniques applied for the cell cortex dataset include (left to right): Gaussian blur, element-wise addition, contrast normalization, simple pixel value addition, gamma adjustment, multiplying pixel values, dropout pixels, adding Gaussian noise, flipping left/right, flipping up/down, cropping, scaling, rotating, translating, and shearing the image. **(b)** The same augmentation techniques as described in a were applied for the spindle dataset. Scale bar: 5 µm.

To perform the segmentation of label-free cell cortex and fluorescently-labeled mitotic spindle, we trained and compared two groups of neural network models referred to as “SpinX-base” and “SpinX-optimized.” The two models differed in annotation quality and the number of epochs, with an increased number for the optimized model (Table S2). Annotations for the base model were created by beginner users (0–2 yr experience in Cell Biology, *N* = 800 cortex and 900 spindle images), whereas annotations for the optimized model were created by expert users (>3 yr experience in Cell Biology, *N* = 1,300 cortex and 1,390 spindle images). For both SpinX-base and SpinX-optimized, data augmentation techniques were carefully selected to artificially increase image variety. For label-free cell cortex images, augmentation was achieved by blurring through Gaussian filtering and contrast normalization (Fig. S4 a). This was performed to improve the robustness of the cell cortex model in segmenting uniform pixel signals within the cytoplasm. Translation, rescaling, rotation, and shearing were added to address the natural variation in cell shape and size (Fig. S4 a). For mitotic spindle images, higher priority was given to image flipping and rotation in order to better emulate spindle dynamics (Fig. S4 b).

**Figure S5.**
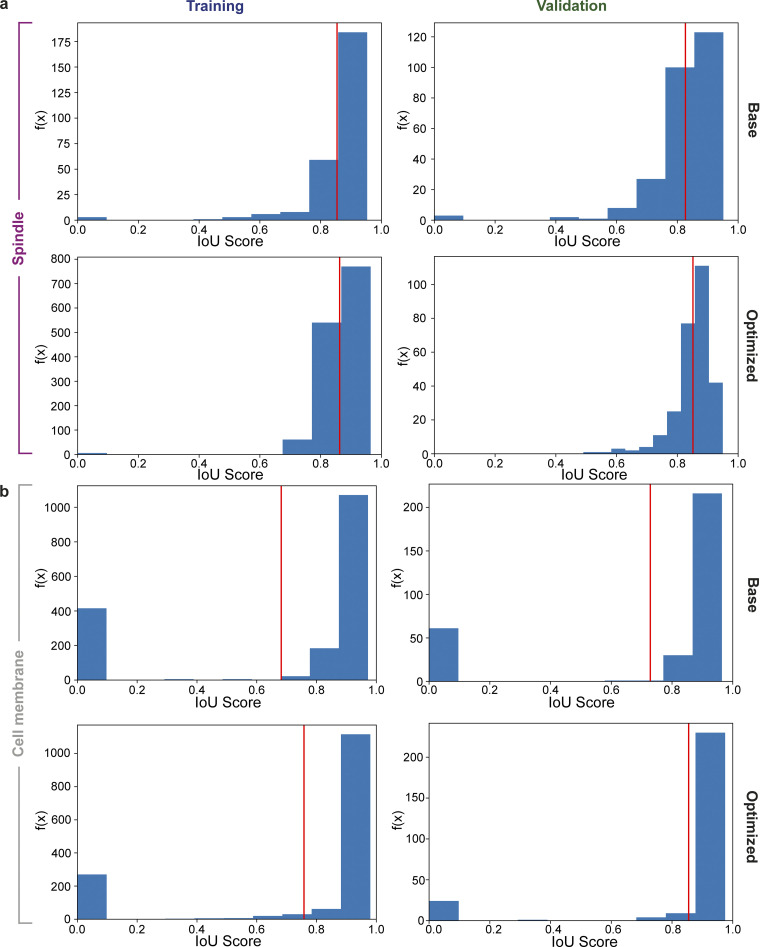
**Computational evaluation of SpinX-base and SpinX-optimized models using the IoU metric. (a)** Histogram of IoU for base and optimized models derived from the spindle training and validation datasets. **(b)** Histogram of IoU for base and optimized models derived from cell membrane training and validation datasets. Red horizontal line indicates mean of IoU (mIoU).

**Figure S6.**
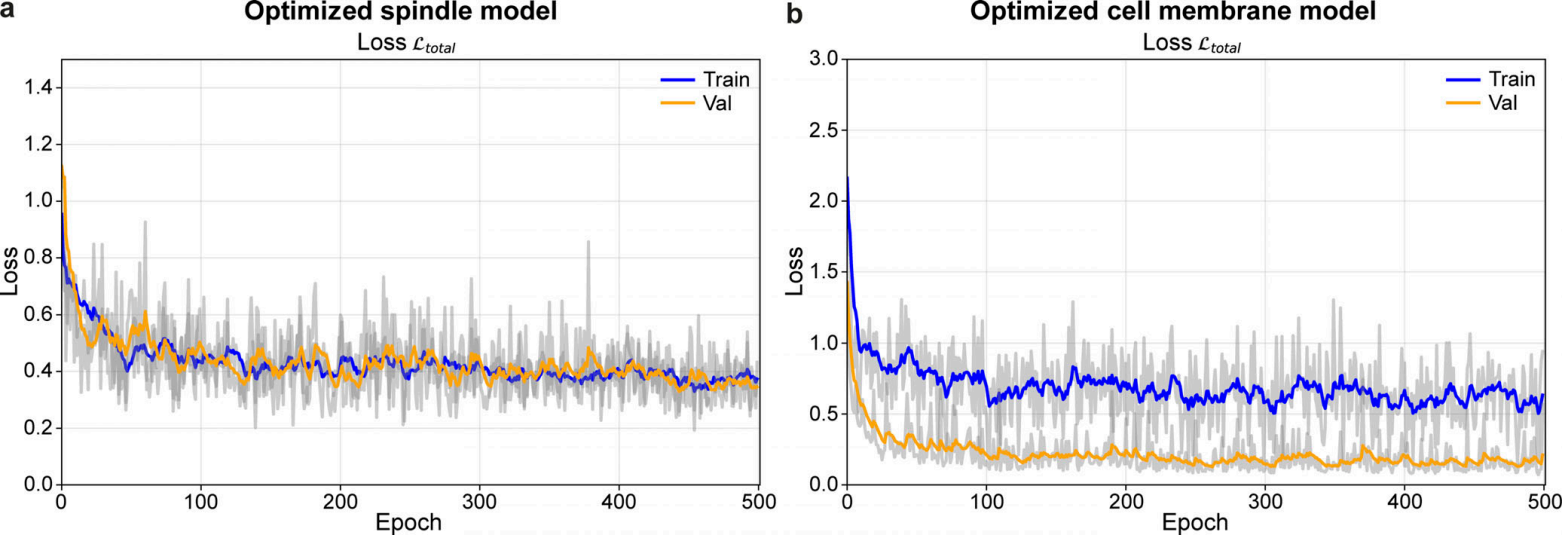
**Computational evaluation of SpinX-base and SpinX-optimized models using the Loss function metric. (a)** Line graphs show declining training (blue) and validation (orange) loss functions for the optimized spindle model. **(b)** Line graphs show declining training (blue) and validation (orange) loss functions for the optimized cell membrane model.

Model performance was examined through metrics, such as mean Intersection over Union (IoU), mean average precision (AP) and Loss function, allowing the evaluation of classification and mask accuracy (Figs. S5 and S6). Comparison of the SpinX-base and SpinX-optimized spindle models suggested an improvement in the ability of SpinX-optimized to generalize, leading to more accurate predictions on unseen data (validation dataset). To assess the extent of correct predictions, we used Average Precision (AP, a metric to indicate how well the model detects the spindle as a whole object), IoU (a metric for which pixels/areas belong to the spindle) and other standard metrics, including loss reduction scores, to evaluate object detection models (He et al., 2018; Minaee et al., 2021; Ronneberger et al., 2015). AP for the validation dataset was greater by 0.053 in the optimized version, along with marginal improvements in AP (0.012) and loss reduction (0.0365) for the training dataset (Figs. S5 and S6; and Table S3). The optimized model has fewer outliers compared to the base model (IoU scores closer to 0 are arising from misclassification), making the optimized model as the preferred model. For the cell cortex model, SpinX-optimized displayed a notably higher mean IoU (0.122) and AP (0.124) than SpinX-base for the validation dataset, suggesting a reduced occurrence of errors during classification and higher accuracy when predicting segmentation masks (Figs. S5 and S6; and Table S3). To gain further insight on how annotation quality affected model performance, we manually examined the annotations of SpinX-base. 44% and 31% of images required re-annotation to precisely outline the boundaries of the cell cortex and spindle, respectively (Table S4). Considering the elevated performance of SpinX-optimized compared to SpinX-base, we concluded that annotation quality and the hyperparameters used to train a Mask R-CNN-based model can greatly affect performance, and so we use SpinX-optimized for subsequent studies.

**Figure 2.**
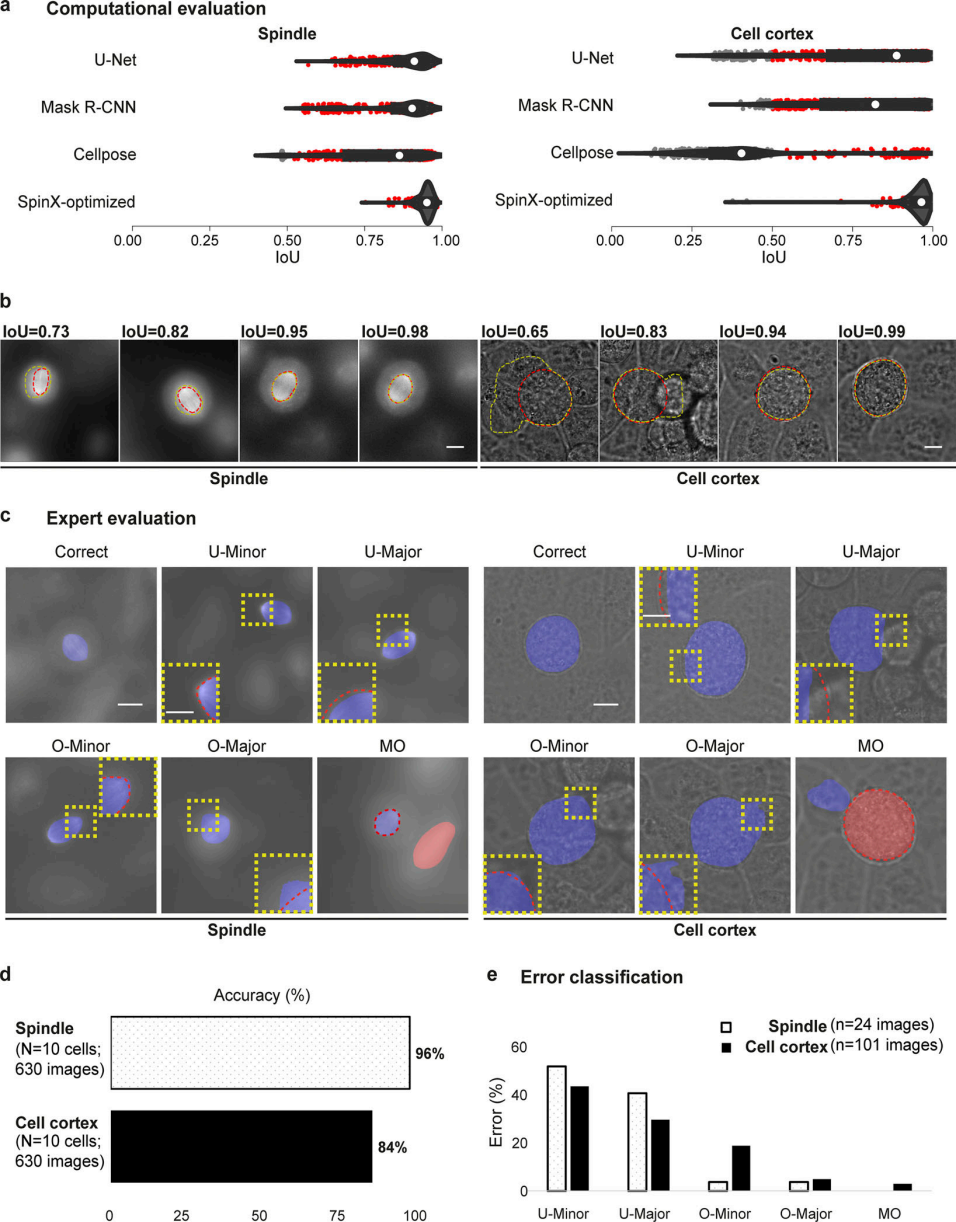
**Computational and manual evaluation of SpinX shows high accuracy for spindle and cell cortex segmentation. (a)** Violin plots show the distribution of IoU scores calculated from predictions with U-Net, Mask R-CNN, Cellpose, and SpinX-optimized. White marker within the box refers to the median, the shaded area refers to the estimated kernel probability density, and the box indicates the interquartile range of the data. Gray and red dots correspond to IoU scores smaller or >0.5, respectively. **(b)** Representative images show a range of different IoU scores calculated between the ground truth (red line) and predicted mask (yellow line) for the spindle (left) and cell cortex (right). Scale bars: 10 µm. **(c)** Representative SpinX prediction images for spindle (left) and cell cortex (right) describing the manual error classification system. Incorrectly segmented images were classified into “under segmentation minor” (U-Minor), “under segmentation major” (U-Major), “over segmentation minor” (O-Minor), “over segmentation major” (O-Major), and “multiple objects with artifacts” (MO). Insets show higher magnification of observed errors (yellow box). The prediction is highlighted by the blue and red overlays with the corresponding ground truth marked by a red dashed outline. Scale bars: 10 µm, 5 µm for inset. **(d)** Bar chart shows SpinX’s final accuracy, manually evaluated, for the spindle (white) and cell cortex (black) models. **(e)** Bar chart shows the proportion of incorrectly segmented images for each error type defined in c without Stage 3 of SpinX. For b, d, and e, *N* = 1,260 images (630 images each for spindle and cell cortex) from 10 3D time-lapse movies across four independent experiments were considered.

**Figure S7.**
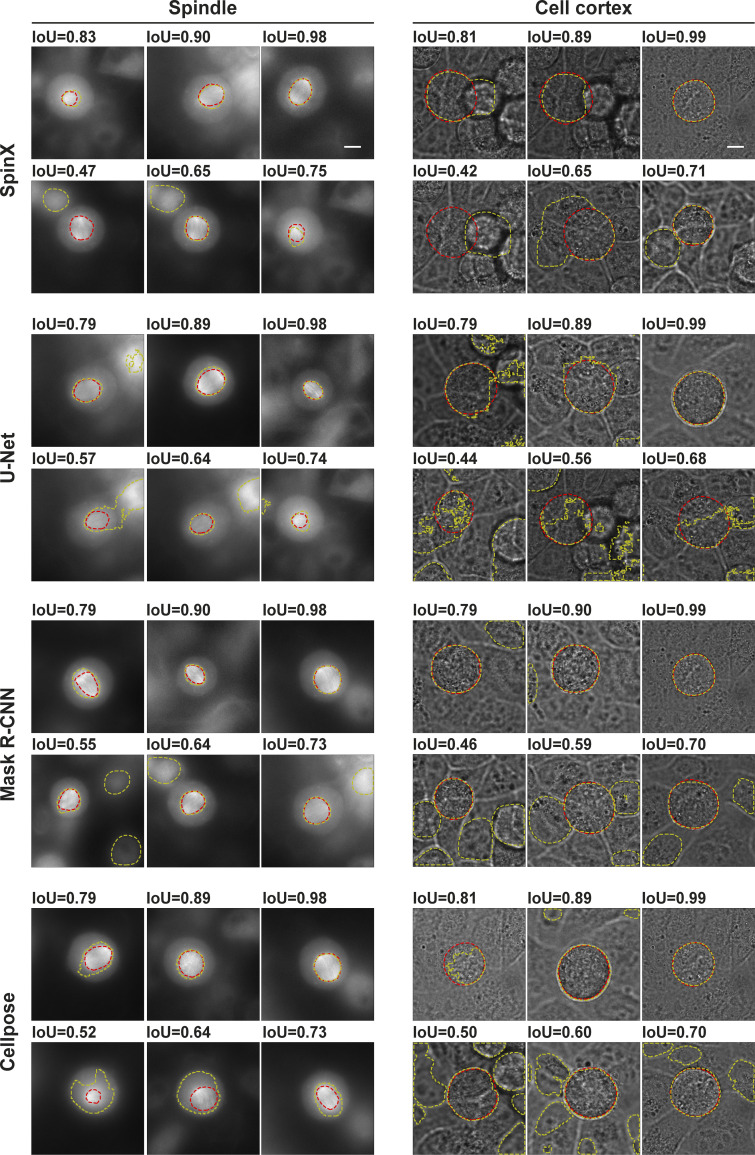
**Example segmentations from different architectures tested with our dataset.** Representative spindle (left) and cell cortex (right) images showing a range of different IoU scores calculated between the ground truth (red line) and predicted masks (yellow line) from the different architectures tested. Scale bars: 10 µm.

Next, we benchmarked the ability of SpinX to perform segmentation through temporally and spatially discontinuous image sequences. For this, we randomly selected a separate set of 10 time-lapse movies and examined model performance on the cell cortex and the spindle. As routinely performed in the DL field (He et al., 2018; Minaee et al., 2021; Ronneberger et al., 2015), we computationally evaluated model performance on the spindle and cell cortex segmentation (*n* = 630 previously unseen images for each model) without (“native” Mask R-CNN) and with (SpinX-optimized) post-processing (Fig. 2, a and b). We also compared the model performance of SpinX-optimized against U-Net, a DL architecture that has been previously used for cell segmentation (Falk et al., 2019; Ronneberger et al., 2015; Stringer et al., 2021). For this, we trained the U-net-based models using the same training datasets (*n* = 1,300 cell cortex model; *n* = 1,390 spindle model; Fig. 2 a). In addition, we used our images to evaluate the currently available pretrained Cellpose models (pretrained nucleus model for spindle and cyto model for cell cortex; Stringer et al., 2021; Fig. 2 a). Model performance was evaluated by matching the predictions of each model to the ground truth masks through the IoU metric (Fig. 2, a and b) as in representative IoU images (Fig. S7). For both spindle and cell cortex segmentation, SpinX outperformed the other three state-of-the-art methods (Fig. 2 a).

**Figure S8.**
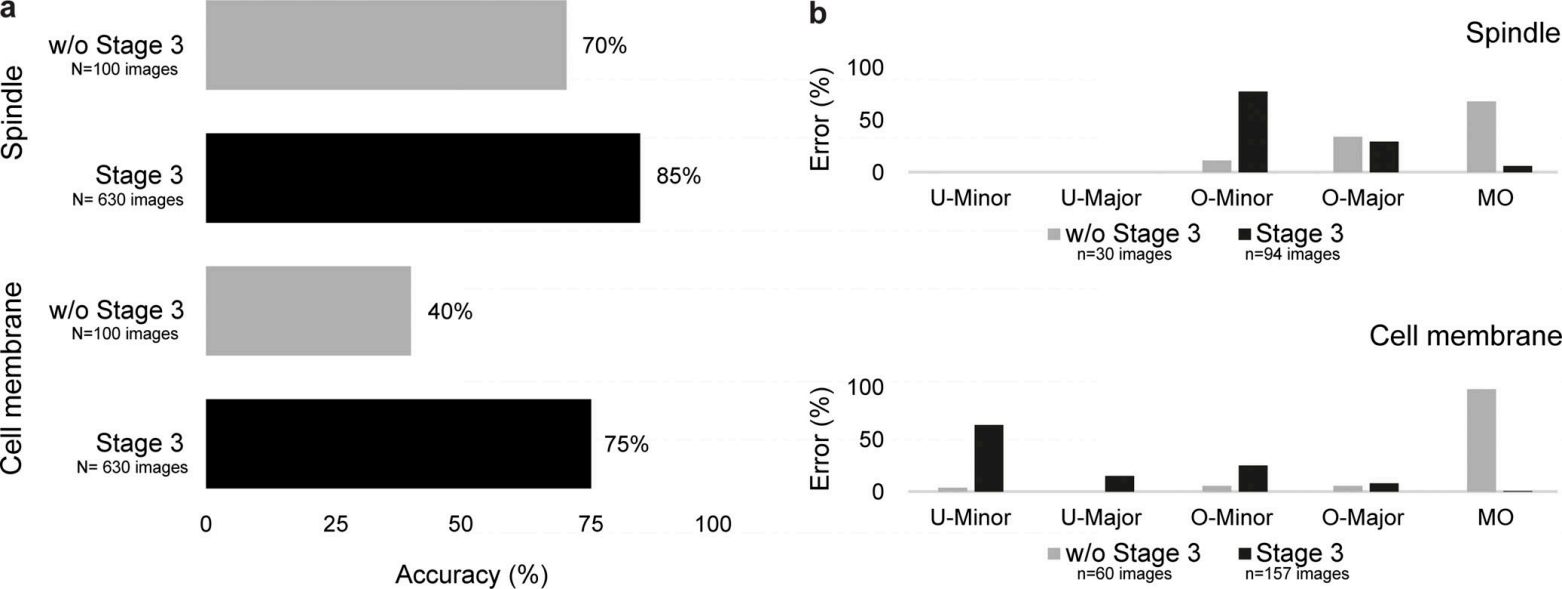
**SpinX Stage 3 evaluation. (a)** Bar chart shows accuracy without (gray) and with Stage 3 (black) of SpinX’s architecture for spindle and cell membrane models. **(b)** Wrongly predicted images from spindle and cell membrane models were further analyzed using our error classification system (described in Fig. 2 c). *n* = 100 randomly selected images from our image pool were used for studies without Stage 3. *n* = 630 images from 10 randomly selected 3D time-lapse movies were used for studies with Stage 3.

As the output of the AI module is directly fed into the 3D modeling module, accurate boundary information is crucial for reliable 3D tracking of objects. Routinely used IoU metrics, although useful for conventional image segmentation purposes, are insufficient for the purpose of spindle tracking because similar IoU scores can reflect different errors in boundary information (Fig. 2 b and Fig. S7). For example, the consequence of errors in boundary information near spindle poles will be far more severe than around spindle walls; similar exceptions would apply for cell cortex boundaries (Fig. 2 b and Fig. S7). Hence, to dissect how the model performance metrics (Fig. 2, a and b) translate into accurate segmentation of the spindles and the cell cortex, we developed an error classification system (Fig. 2 c). We assessed five types of distinct errors: undersegmentation minor (U-Minor), undersegmentation major (U-Major), oversegmentation minor (O-Minor), oversegmentation major (O-Major), and multiple objects (MO; Fig. 2 c). We benchmarked the SpinX-base models on a large dataset of 630 images with Stage 3 of SpinX’s architecture activated (Fig. 1 b), which significantly increased the overall accuracy by 35% for the cell cortex model, and 15% for the spindle model (Fig. S8). We could confirm that the enhanced accuracy was mainly due to the elimination of wrongly predicted images categorized within the “MO” class (Fig. S8). Utilizing the SpinX-optimized models (for the same set of 630 images) led to an even greater increase in overall accuracy when compared to SpinX-base—11% for the spindle model and 10% for the cell cortex model, whereby most errors were found under the “U-Minor” class for both models (Fig. 2, d and e). In summary, following different optimizations, SpinX’s final accuracy reached 85% for the cell cortex model and 96% for the spindle model (Fig. 2 d).

### Generalization of SpinX to different spindle markers, cell lines, and distinct imaging systems

**Figure 3.**
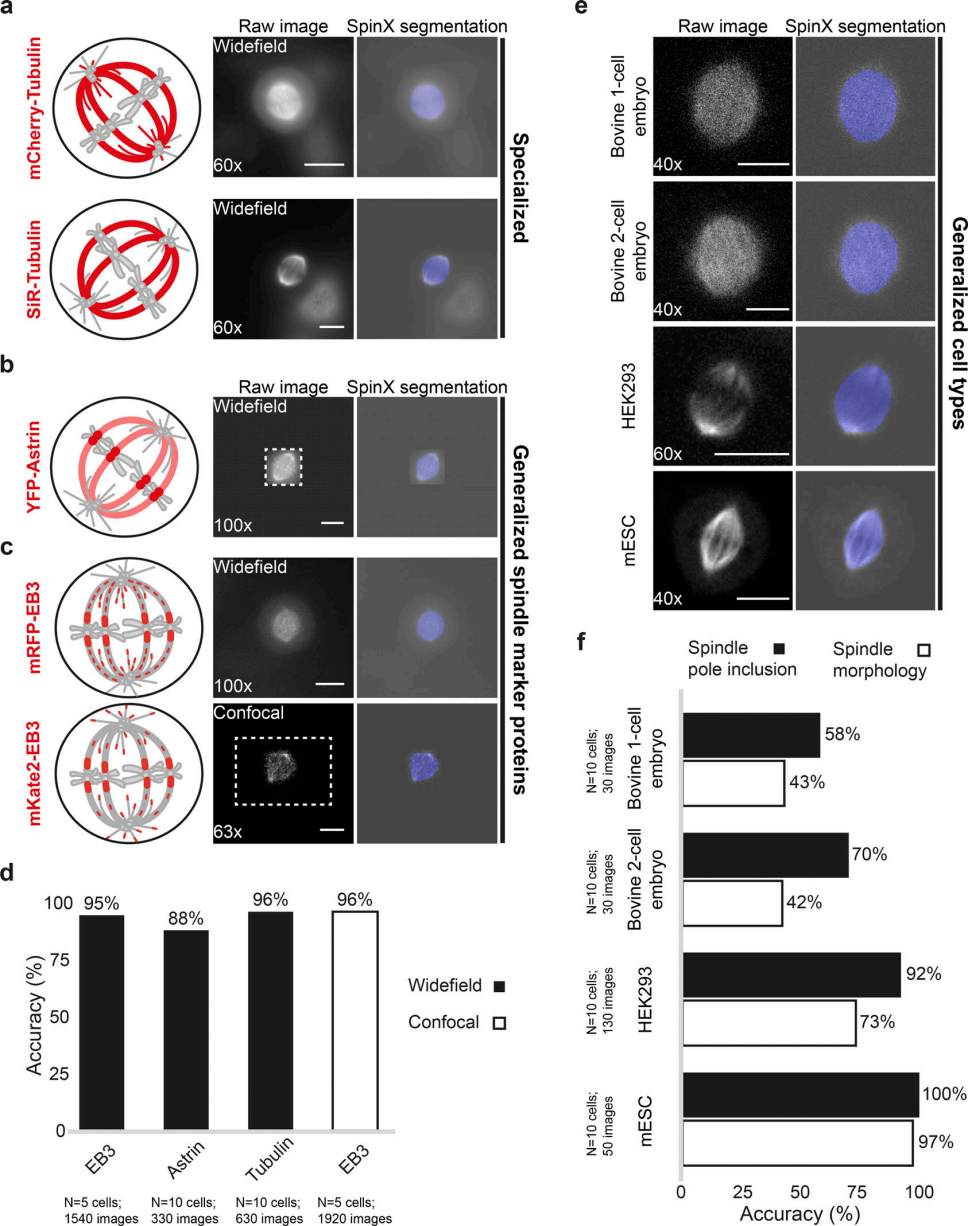
**Generalization of SpinX to segment images of new marker proteins and cell types acquired using distinct microscopes. (a)** Representative images and SpinX segmentation output of mCherry-Tubulin and SiR-Tubulin datasets used to train the spindle model (specialized). **(b and c)** Representative images and SpinX segmentation output of YFP-Astrin (b), mRFP-EB3 and mKate2-EB3 (c) datasets used to assess the extent to which the spindle model can be generalized. The dashed box shows the original image border (YFP-Astrin and mKate2-EB3 datasets were padded for analysis). Cartoons in a, b, and c show differing localization patterns (red) of spindle marker proteins. Images acquired using a widefield or higher resolution confocal microscope are highlighted, where the associated objective used is indicated. **(d)** Bar graph shows SpinX’s segmentation accuracy of spindles labeled using mRFP-EB3, YFP-Astrin, and Tubulin (mCherry-Tubulin and SiR-Tubulin combined) acquired with a widefield microscope, and mKate2-EB3 using a confocal microscope as indicated. Accuracy was manually scored by experts using the error classification system indicated in Fig. 2, c and e. **(e)** Representative images and SpinX segmentation output of bovine one-cell embryo, bovine two-cell embryo, HEK293, and mESC datasets used to assess the extent to which the spindle model can be generalized to spindles from other cell types. Images provided by Kletter et al. (2022) were acquired using a confocal microscope, where the associated objective used is highlighted. **(f)** Bar graph shows SpinX’s segmentation accuracy of spindles from other cell types, segregated into either the “Spindle pole inclusion” or “Spindle morphology” category. Categories were created based on the error classification system outlined in Fig. 2, whereby “Spindle pole inclusion” includes both images classified as “Correct” and “U-minor,” while “Spindle morphology” includes only images classified as “Correct.” Spindles with a visible midzone were chosen. Accuracy was manually scored by experts. mRFP-EB3 *N* = 5 cells, 1,540 images; YFP-Astrin *N* = 10 cells, 330 images; SiR/mCherry-Tubulin *N* = 10 cells, 630 images; mKate2-EB3 *N* = 5 cells, 1,920 images; bovine one-cell embryo *N* = 10 cells, 30 images; bovine two-cell embryo *N* = 10 cells, 30 images; HEK293 *N* = 10 cells, 130 images; mESC *N* = 10 cells, 50 images. Scale bars: 10 µm.

As neural network models that accurately segment “unseen” types of data signify longevity and wider applicability, we examined the generalization capacity of the SpinX framework. Our training dataset consisted of spindles labeled using either mCherry-Tubulin or SiR-Tubulin dye, markers of tubulin subunits (Fig. 3 a) which are responsible for assembling and disassembling microtubules of the mitotic spindle (reviewed in Tamura and Draviam 2012). To examine the extent to which SpinX can be generalized, we evaluated the accuracy of SpinX in detecting spindles in time-lapse movie datasets where two different fluorescent marker proteins were fused to two distinct microtubule-binding proteins. First, we tested image datasets of cells expressing YFP-tagged Astrin, a microtubule-wall binding protein that can be found at the chromosome–microtubule attachment site soon after the tethering of microtubule ends to chromosomes (Conti et al., 2019; *n* = 330 images from 10 cells; Fig. 3 b). Model evaluation was carried out by an expert user through manual binary classification of either “correct” or “incorrect” prediction. Expert evaluation showed that SpinX can successfully segment spindles in movies of YFP-Astrin expressing cells with an 88% accuracy (Fig. 3 c). The images in the YFP-Astrin dataset were not complete images of the entire mitotic cell but instead cropped images encompassing the spindle structure alone, requiring padding (see Materials and methods) to allow segmentation through SpinX. Next, we tested image datasets of cells expressing mRFP-tagged End-Binding 3 (EB3), a growing microtubule-end binding protein (Komarova et al., 2005) that can be found at the chromosome–microtubule attachment site and spindle poles (Tamura and Draviam 2012; *n* = 1,540 images from 5 cells; Fig. 3 b). In addition to widefield images, we extended our evaluation to high-resolution confocal images of cells expressing mKate2-EB3 (Fig. 3 b; *n* = 1,920 images from 5 cells). Expert evaluation showed that SpinX is equally successful in segmenting spindles of EB3 marker expressing cells in both widefield and confocal microscopy images, with a 95% and 96% accuracy, respectively (Fig. 3, c and d). To determine the extent to which SpinX can successfully segment spindles in images of new cell types and embryos, and images acquired using different microscope systems, we used images generated by others and made available as Spindle3D datasets (Kletter et al., 2022). In spindles of different cell types, mESC and HEK293, spindle pole inclusion was successful 100% and 92% of the cases, respectively, despite being imaged using different imaging systems; however, this segmentation efficiency was reduced in large spindles of bovine embryos (Fig. 3, e and f). Nevertheless, in cultured cells, widefield and confocal images of spindles were equally well segmented using SpinX (Fig. 3, d and f). Thus, the successful segmentation of EB3 or Astrin protein marker labeled spindles, and Tubulin labeled spindles in different cell types and distinct microscopy systems demonstrate a striking generalization capacity of the SpinX framework for a variety of spindle markers, cell types, and microscopy methods.

### Modeling to quantify 3D spindle movements relative to the cell cortex

**Figure 4.**
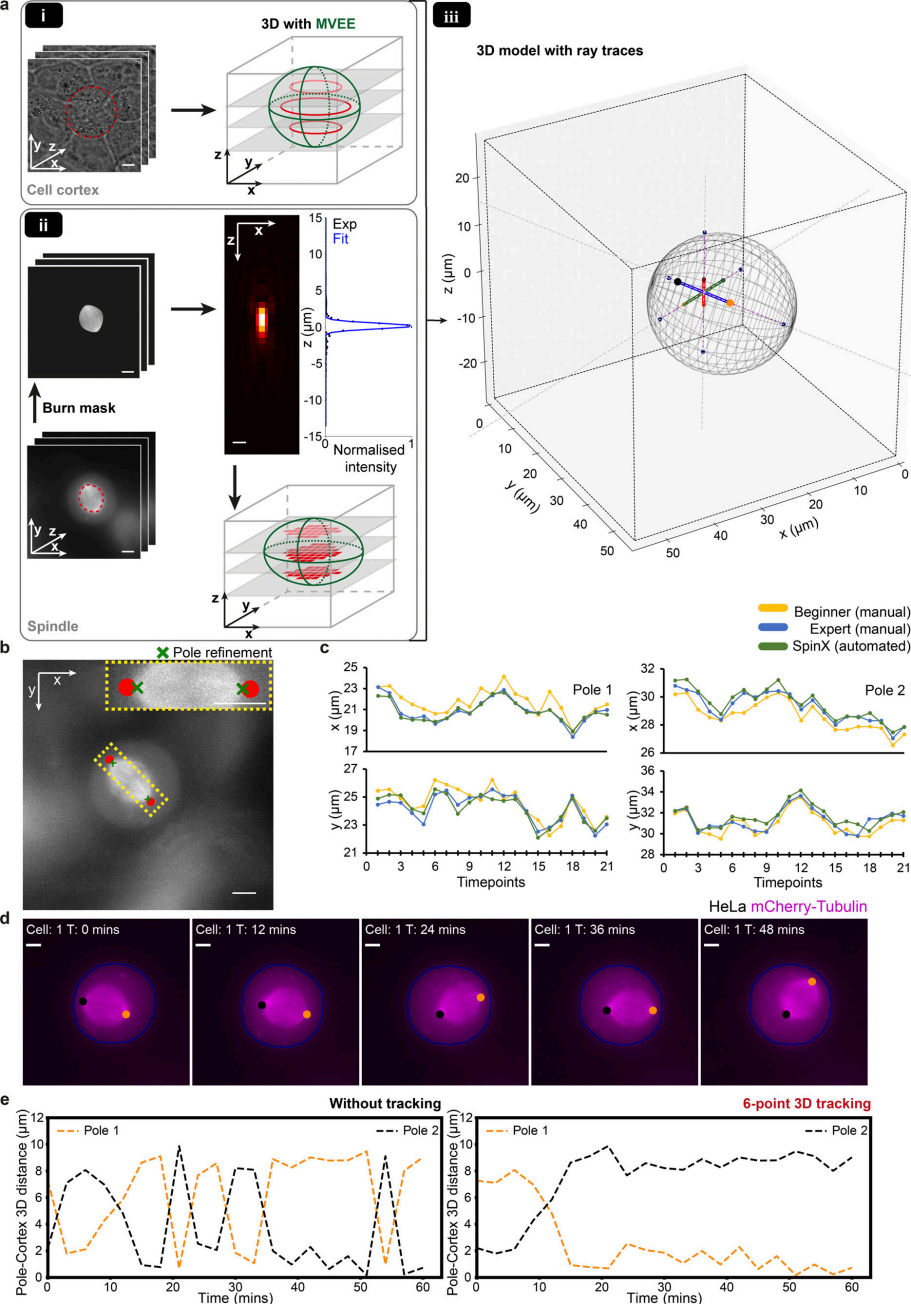
**3D reconstruction and modeling for time-resolved analysis of spindle-cortex interaction changes. (a)** (i) Representative time-lapse images of a mitotic cell (left) with the corresponding outlined masks (red dashed line) predicted with SpinX’s AI module. SpinX utilizes *z*-slices of cell cortex boundaries (red, transparent and inner rings) to reconstruct the 3D shape of the cell via Minimum Volume Enclosing Ellipsoidal fit, MVEE (green, dotted outer rings). (ii) Representative time-lapse images of a spindle with the corresponding outlined masks (red dashed line) predicted with SpinX’s AI module. Merging masks with raw images (burn) removes non-spindle signals. PSF was generated and fitted (blue line) to map intensity fluctuations with changes in axial positions for each pixel (red) belonging to the spindle. To reconstruct the 3D structure of the spindle, MVEE (green) was applied. (iii) The 3D plot shows the complete model. The cell cortex is represented by the polygon mesh in gray with the spindle principal axes, which correspond to spindle height (red), width (green), and length (blue). The large orange and black dots at the ends of the length axis correspond to the spindle’s individual poles, while the smaller dots correspond to the ends of the spindle height (red-filled) and width (green-filled) axes. Ray traces from the spindle poles are represented by dashed gray lines, and their intersection points are marked as dark blue dots on the cortical mesh. The pole-cortex distance is outlined in magenta. **(b)** Demonstration of pole refinement in SpinX. Spindle pole estimations without and with pole refinements are indicated by red dots and green crosses, respectively. **(c)** Representative line plots show x and y coordinate changes of a spindle tracked through time, for its poles 1 and 2, measured either manually by a beginner (yellow), expert (blue), or automatically with SpinX (green). **(d)** Representative max projection time-lapse images of a HeLa cell expressing mCherry-Tubulin. Orange and black dots represent spindle poles 1 and 2, respectively. The cell outline (blue) was extracted from the predicted segmentation mask of cell membrane by SpinX’s AI module. **(e)** Line plots show individual pole-cortex 3D distance measurements computed from d for pole 1 (orange) and pole 2 (black) without and with 3D 6-point tracking, respectively. Scale bars: 5 and 1 µm for PSF in b.

**Figure S9.**
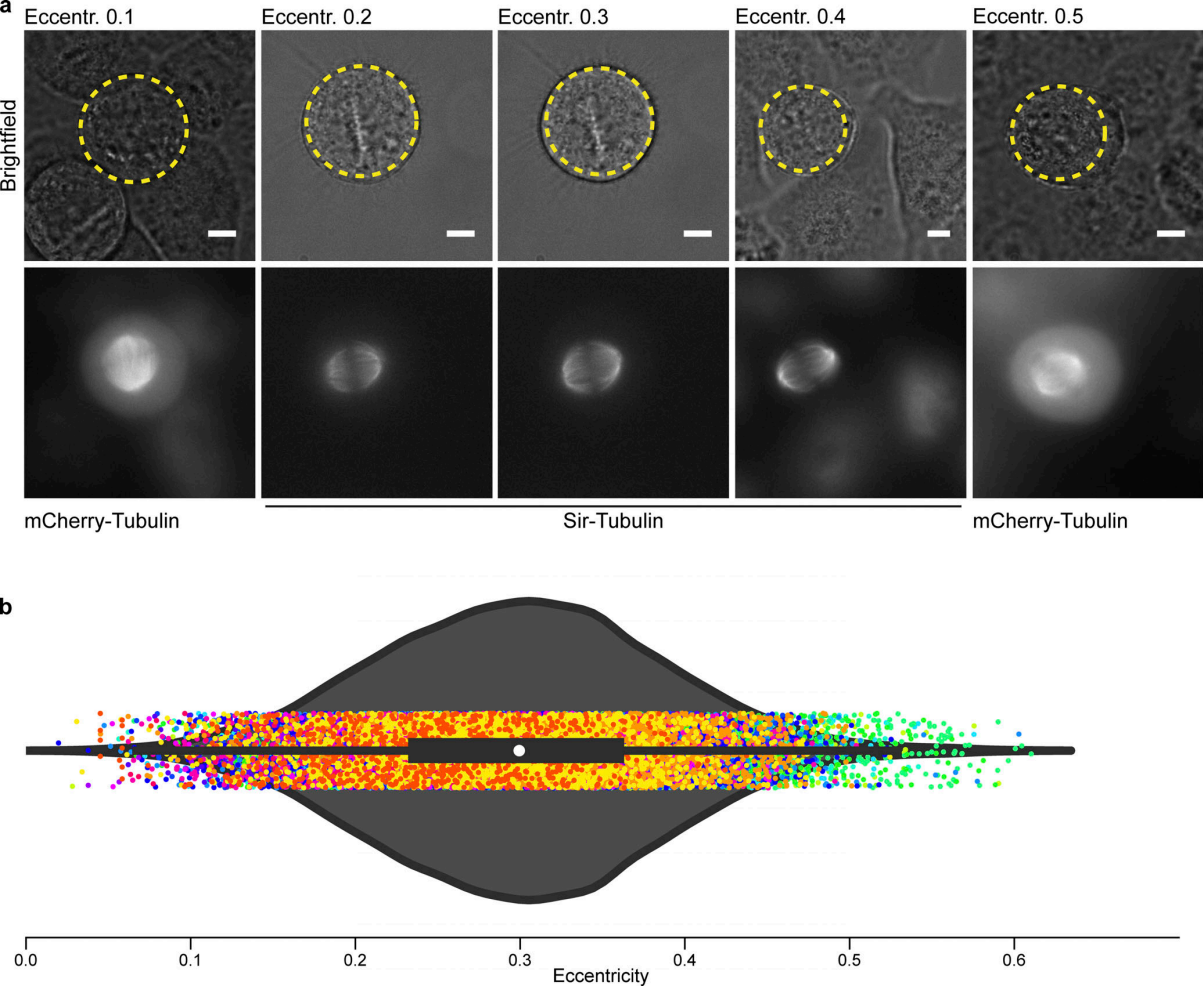
**Cell cortex is not fully circular. (a)** Representative brightfield images (first row) of HeLa cell lines expressing mCherry-Tubulin or Sir-Tubulin. Cells were selected based on measurements of eccentricity. Intact spindles of the individual cells are represented in the second row. Circular shape with eccentricity = 0 is highlighted in yellow. **(b)** Violin plot shows distribution (dots) of eccentricity across 96 3D live-cell movies from 16 experiments. White marker within the box refers to the median, the shaded area refers to the estimated kernel probability density and the box indicates the interquartile range of the data. Measurements from the same dataset share the same color. Scale bar: 5 µm.

Reconstructing a 3D spindle structure and cell cortex from 2D slices is a significant challenge in part due to missing information between the *z*-steps. To track spindle movements with reference to the cell cortex, we used the cell cortex prediction mask from SpinX’s AI module (Figs. 1 and 2) to reconstruct the 3D shape of each individual cell (Fig. 4 a). Although mitotic cells generally assume a distinct spherical shape (Cadart et al., 2014), measuring the eccentricity of cell cortex segmentation masks of 96 cells (Fig. S9), yielded a median value of 0.3 (a value of 0 being a perfect circle) suggesting that using an ellipsoidal rather than a spherical shape may lead to a more reliable 3D reconstruction.

**Figure S10.**
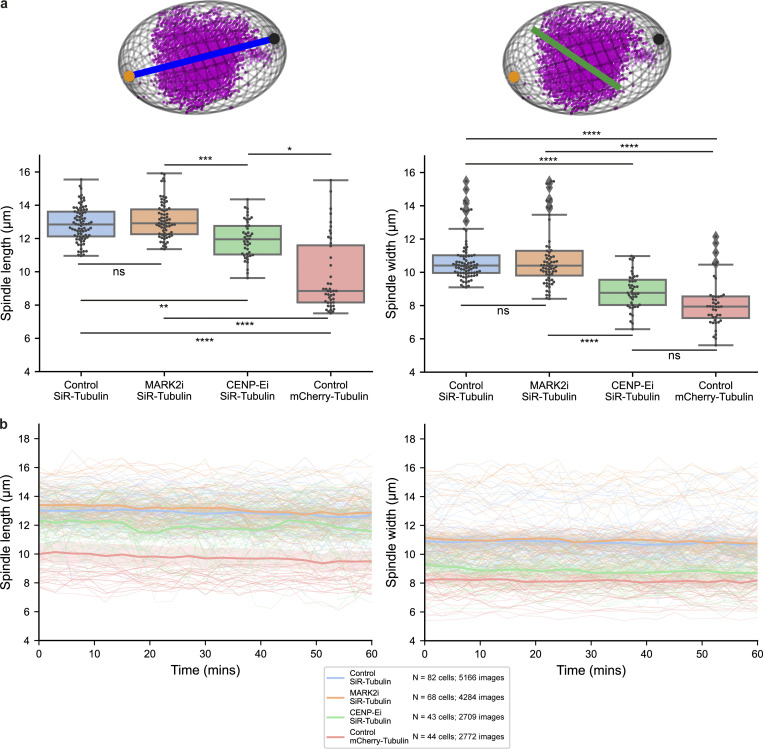
**Spindle length and width measurements computed through SpinX. (a)** Box plots quantifying HeLa SiR-Tubulin and mCherry-Tubulin-labeled spindles length (left) and width (right) as computed through SpinX’s modeling module. Schematics indicate spindle length (blue line) and width (green line) with respect to the detected spindle poles (black and orange dots). **(b)** Line plots quantifying mean (bold) HeLa SiR-Tubulin and mCherry-Tubulin-labeled spindles length (left) and width (right) across time as computed through SpinX. Thinner lines show measurements from individual cells. Shaded area shows 95% confidence interval. For both a and b, Control (DMSO-treated) SiR-Tubulin *N* = 82 cells, 5,166 images; MARK2i SiR-Tubulin *N* = 68 cells, 4,284 images; CENP-Ei SiR-Tubulin *N* = 43 cells, 2,709 images; Control mCherry-Tubulin *N* = 44 cells, 2,772 images. Statistical significance determined with Kruskal-Wallis H test and a post-hoc Dunn’s test with Bonferroni correction: ****P < 0.0001; ***P < 0.001, **P < 0.01, *P < 0.05, ns (non-significant). Non-normality was confirmed with a Shapiro–Wilk test.

**Figure S11.**
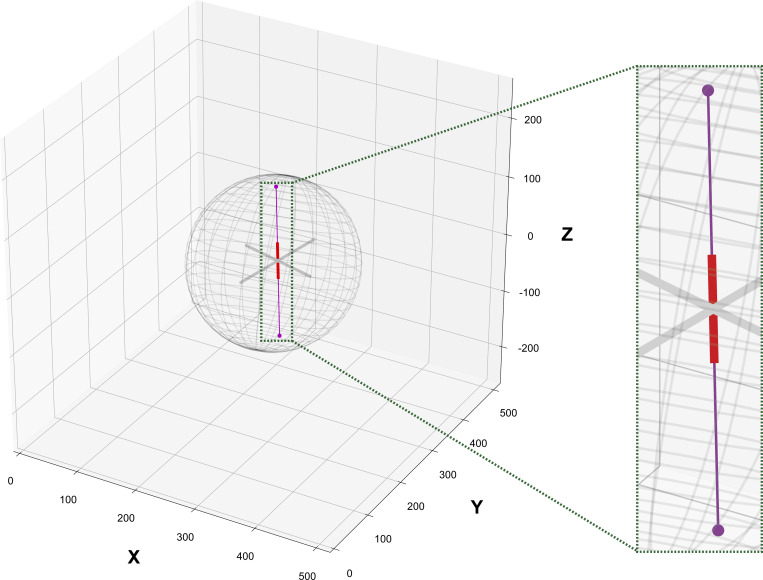
**Analytical solution for 3D Ray-tracing.** Mathematically applying the analytical solution (as described in Materials and methods) results to a connecting line (magenta) of two intersection points (i.e., points of contact between the spindle principal axis’ endpoints and the cell surface) that overlaps with the spindle height axis (red), hence allowing the calculation of pole-cortex distances in 3D.

**Figure S12.**
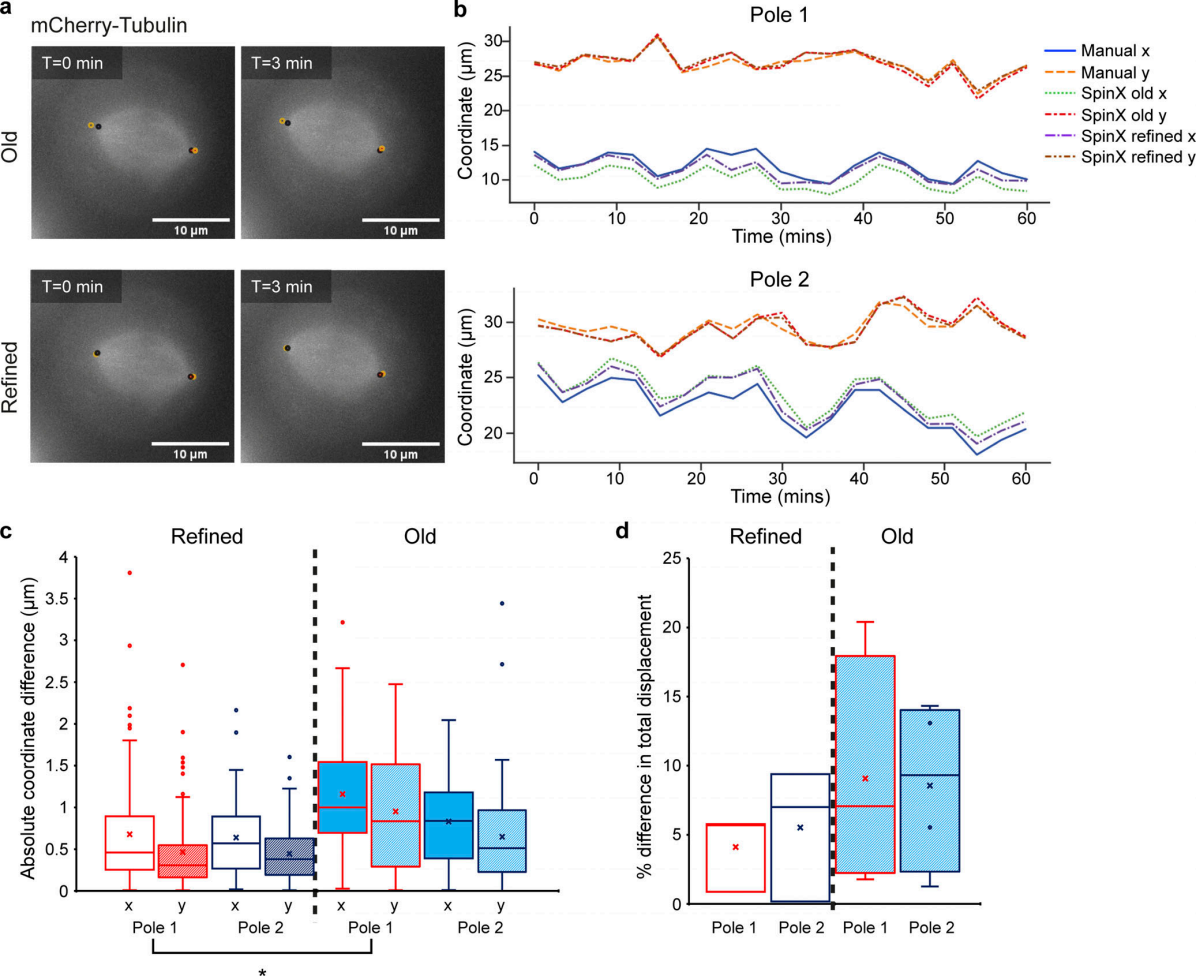
**SpinX’s refined algorithm for recording pole positions is more reliable compared to its previous iteration. (a)** Representative maximum projection images of the mitotic spindle in a HeLa mCherry-Tubulin cell showing pole positions as recorded from SpinX’s old (top) and refined (bottom) algorithm. Black circles correspond to the poles recorded by manual analysis, while orange circles correspond to the poles recorded by SpinX. Pole alignment was kept consistent throughout measurements i.e., red-filled circles correspond to pole 1, whereas blue-filled circles correspond to pole 2. T corresponds to the time at which the image was taken. Scale bar = 10 μm. **(b)** Representative traces of the x-y coordinates of the poles of a single cell across time as recorded from manual analysis, and SpinX’s old and refined algorithm (Pole 1—top; Pole 2—bottom.). **(c)** Box plots quantifying the absolute coordinate (x-y) difference between manual analysis and either refined (left) or old (right) SpinX for both Poles 1 and 2. *N* = 4 cells, each cell consisting of 21 measurements for each pole. Kruskal–Wallis H test + post-hoc Dunn’s test with Bonferroni adjustment: *P <0.05 Pole 1 refined vs. Pole 1 old. **(d)** Box plots quantifying the % difference in spindle pole total displacement between manual analysis and either the refined (left; *N* = 3 cells) or old (right; *N* = 4 cells) versions of SpinX. The comparison between manual analysis and SpinX was performed based on x-y coordinates alone, but it should be noted that SpinX performs 3D reconstruction, thus predicting x-y positions while taking into consideration the *z*-position.

We reconstructed the 3D structure of the cell cortex using label-free brightfield images by applying a Minimum Volume. Enclosing Ellipsoid fit (MVEE) on the boundary pixel coordinates extracted from the prediction mask of the cell cortex (Fig. 4, a i). To reconstruct the 3D structure of the spindle using fluorescent images, we took advantage of the point-spread function (PSF) of our imaging system. The PSF enabled the estimation of the *z*-coordinates of spindle-associated pixels, which were subsequently used to reconstruct the spindle’s 3D structure by applying the MVEE (Fig. 4, a ii). To investigate the integrity of the reconstructed 3D spindle structure, we compared spindle length and width measurements using 3D pole positions (Fig. S10). We observed spindle width and length were consistent with previous volumetric morphometric studies (Kletter et al., 2022). To capture spindle movement relative to the cell, we measured spindle pole-to-cortex distances (Fig. 4, a iii purple line). For this, we modeled 3D ray traces where we analytically identify the intersection points between the spindle’s principal (pole-to-pole) axes and the rounded cell cortex. Thus the line that passes the two intersection points (Fig. 4, a iii dark blue dots) at the cortex, will pass through the spindle axis of interest as well (see Materials and methods; Fig. 4, a iii dashed gray and purple line; and Fig. S11 purple line). This required that the spindle poles are precisely identified, and hence we benchmarked the extent to which MVEE could accurately identify spindle length (the long-axis of the ellipsoid). We observed that due to the intrinsic structure of the spindle, MVEE tends to overestimate the spindle length, which, in turn, alters the predicted spindle pole position (Fig. 4 b). This bias accounted for a median spindle pole displacement of 0.5–1 µm in SpinX, compared to manual analysis, which is a 5–6% difference in total spindle displacement (*N* = 4 cells, 84 instances; Fig. S12). This could be recalculated by extracting 3D coordinates along the spindle’s pole-to-pole axis to identify the first and last occurrence of high-intensity values that were then assigned as refined spindle pole locations (Table S1). Comparing spindle pole locations obtained either with SpinX or manually (both beginners and experts) confirmed that SpinX’s measurements with the pole refinement algorithm (Table 1) closely match with the positions tracked manually by an expert while outperforming manual assessment by a beginner (Fig. 4 c).

### Tracking of 3D spindle movements through time

To study temporal changes in the spindle’s 3D position, we implemented a six-point tracking algorithm based on the *k*-NN algorithm. The six points represent the endpoints of the three principal axes of the ellipsoid which correspond to the spindle height, width, and length axes. By assigning the smallest Euclidean distance to Pole 1 and not Pole 2, we ensured the correction of falsely assigned pole identities through time (refer to Materials and methods). To test how frequently corrections have to be assigned, we analyzed 10 randomly selected time-lapse movies. Correction with *k*-NN was required for around half of the time for spindle width and length axes, and one-third for spindle height axis (Table S5). To evaluate the impact with and without tracking, we measured the 3D distances from each spindle axis to the cell cortex (Fig. 4 d). The spindle pole-to-cell cortex (pole-cortex) 3D distance was obtained by computing ray traces. We confirmed that consistent pole assignments with the tracking algorithm enabled accurate measurements of spindle pole positions through time (Fig. 4 e). Thus, changes in spindle pole to cell cortex distances, as a measure of spindle displacement, could be tracked in 3D through time (Video 1).

**Video 1.** **Spindle and cell cortex tracking in 3D with SpinX.** Video shows composite time-lapse movies of spindle movements in a metaphase HeLa cell expressing mCherry-Tubulin (spindle marker in purple). Raw time-lapse image of mCherry-Tubulin labeled spindle (purple) merged with corresponding brightfield (gray) image of the cell (top-left) or SpinX’s AI module predicted cell cortex outlined in blue (top-middle). Top-right, movie of SpinX’s mathematical object modeling output showcasing spindle pole movements in 3D through time within the metaphase cortex, with an inset displaying the 3D reconstructed spindle; bottom, animated graph highlighting the dynamic change in pole-cortex distances of the two spindle poles as tracks through time.

### SpinX enables the segregation of spindle movements in three distinct dimensions

**Figure 5.**
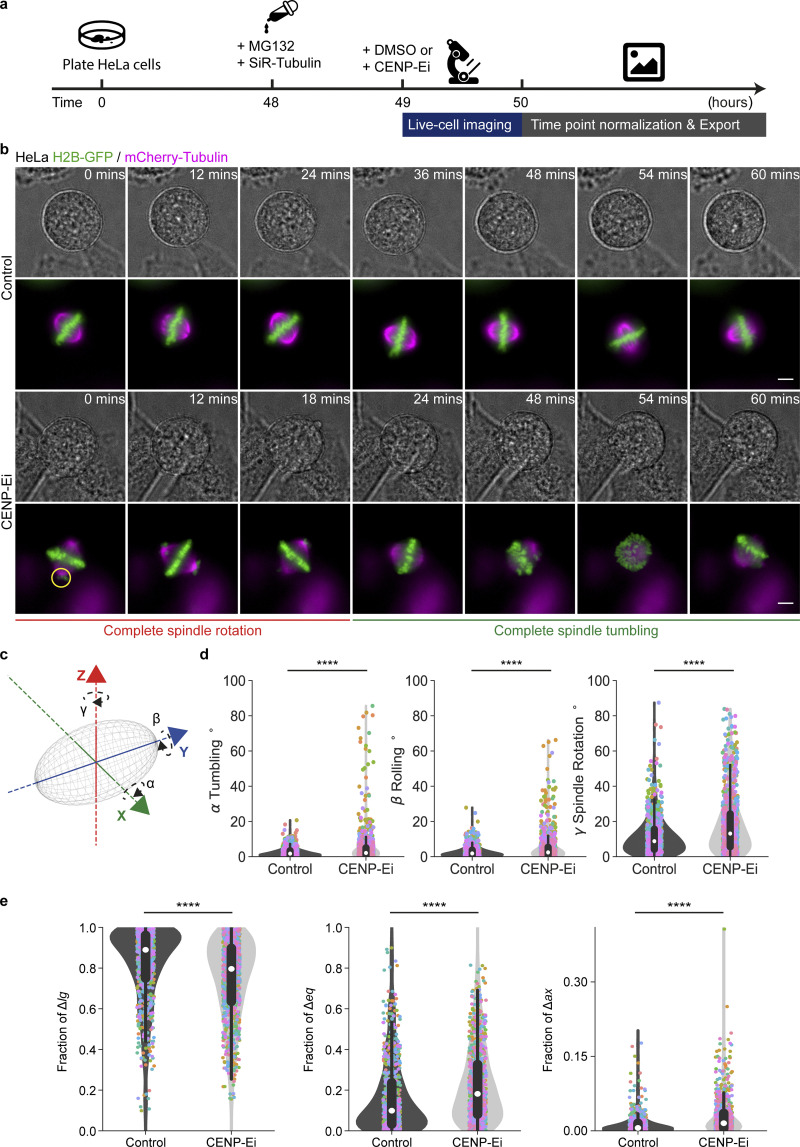
**CENP-Ei promotes rotational movements in 3D. (a)** Experimental regime. HeLa cells were exposed to MG132 (10 µM) 1 hour before imaging and SiR-Tubulin (100 nM) before imaging. Either DMSO (control) or 30 nM CENP-E inhibitor (CENP-Ei) as indicated were added during imaging. **(b)** Representative maximum projection live-cell images of a HeLa cell expressing H2B-GFP (green) and stained with SiR-Tubulin dye (magenta) for 60 min prior to imaging. Cells were imaged for over 1 h with images taken every 3 min. Yellow circles indicate uncongressed chromosomes. **(c)** Cartoon shows a 3D spindle (gray) with the corresponding rotation angles *α* (spindle tumbling), *β* (spindle rolling), and *γ* (spindle rotation) along its principal spindle axes *x,y,z*, respectively. **(d)** Violin plots show 3D angle distribution for *α* spindle tumbling, *β* rolling, and *γ* rotation. Corresponding colored dots represent measurements from all time points of the same cell. The white marker within the box refers to the median, the shaded area refers to the estimated kernel probability density, and the box indicates the interquartile range of the data, respectively. **(e)** Violin plots show fractions of longitudinal, equatorial, and axial spindle displacement. Corresponding colored dots represent measurements from all time points of the same cell. The white marker within the box refers to the median, the shaded area refers to the estimated kernel probability density, and the box indicates the interquartile range of the data, respectively. Statistical significance was determined by Generalized Linear Model (GLM) and Mann–Whitney U test (in d and e) after a pre-analysis of the underlying distribution. *N* = 38 control and *N* = 43 CENP-Ei cells from three experiments. Scale bars: 5 µm.

Inhibition of CENP-E motor protein is known to interfere with the formation of mature kinetochore–microtubule attachments (Kapoor et al., 2006; Shrestha and Draviam 2013) and metaphase chromosome misalignment that in turn promotes excessive spindle movements (Kiyomitsu and Cheeseman 2012). Whether CENP-E inhibitor-treated cells exhibit spindle movement in one direction more than the other is not known. Since SpinX software could readily allow us to separate spindle movements in three dimensions, we tracked spindle movements using time-lapse movies of MG132-treated metaphase HeLa cells expressing H2B-GFP and mCherry-Tubulin exposed to CENP-E inhibitor (CENP-Ei, GSK-923295; Fig. 5 a). Unlike control cells, those treated with CENP-Ei show excessive spindle movements in 3D and unaligned polar chromosomes as expected (Fig. 5 b). We split the 3D movements of the spindle into three groups: spindle tumbling (*α*), spindle rolling (*β*), and spindle rotation (*γ*) movements (Fig. 5 c).

**Figure S13.**
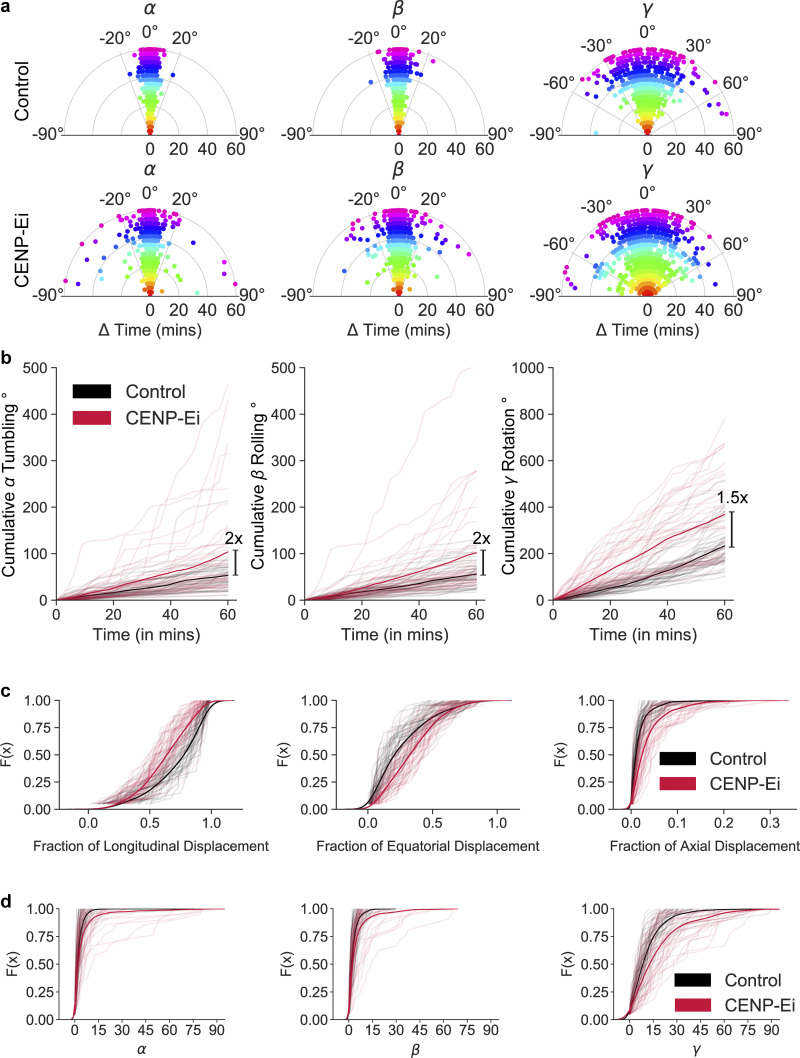
**CENP-E inhibition promotes 3D spindle rotation. (a)** Polar scatter plot shows spindle rate of rotational movement (in degrees) through time. Measurements at each time point are highlighted with a unique color. *α*, *β*, and *γ* angles refer to spindle tumbling, rolling, and rotation. **(b)** Line plots show average cumulative rate of rotation for spindle tumbling (*α*), spindle rolling (*β*), and spindle rotation (*γ*) against time for control (black) and CENP-Ei (red) inhibited cells, respectively. Fainted lines correspond to individual spindle trajectories. **(c)** Empirical Cumulative Distribution Function plots show cumulative fraction for longitudinal, equatorial, and axial displacement for control (black) and CENP-Ei (red) inhibited cells, respectively. Fainted lines correspond to individual spindle trajectories. **(d)** Empirical Cumulative Distribution Function plots show cumulative mean angle values for spindle tumbling (*α*), spindle rolling (*β*), and spindle rotation (*γ*) for control (black) and CENP-Ei (red) cells, respectively. Fainted lines correspond to individual spindle trajectories. Statistical significance was determined by GLM and Kruskal–Wallis test (in c and d) after pre-analysis of the underlying distribution. *N* = 38 control and *N* = 43 CENP-Ei cells from three experiments.

In control metaphase cells, the median angle changes within 3 min are similar between spindle tumbling (*α* median = 1.7°) and rolling (*β* median = 1.6°) and are relatively small compared to spindle rotation movement (*γ* median = 8.8°). In contrast, following CENP-Ei treatment, cells show a significant increase in spindle tumbling (*β* median = 2.4°), spindle rotation (*γ* median = 13.1°), and spindle rolling (*β* median = 2.1°). The increase in spindle rolling upon CENP-Ei treatment has not been reported before (Fig. 5 d). The Empirical Cumulative Distribution Function (ECDF) at 0.75 percentile of the data further highlights the significant increase in spindle tumbling (3.3° to 4.9°), spindle rolling (3.6°–5.2°), and spindle rotation (14.9°–23.8°; Fig. S13 d). Thus quantitative analysis of 3D spindle movements using SpinX reveal that CENP-Ei increases the tendency of the spindle to move both parallel and perpendicular to the substratum.

Separate from tumbling or rotational movements, the mitotic spindle is known to undergo longitudinal oscillation along the pole-to-pole axis (Corrigan et al., 2013; Kiyomitsu and Cheeseman 2012). SpinX analysis showed that upon CENP-E inhibition, spindles show a reduction in the fraction of longitudinal movement, while the fraction of equatorial and axial movements are both increased (Fig. 5 e). Analyzing the distribution for each decomposed movement with the Shapiro–Wilk Test (P < 0.00001) confirmed the presence of data skewness where the fraction of longitudinal movement is the strongest followed by equatorial and axial movement in control and CENP-Ei cells. At the 0.75-percentile of the data, the fraction of longitudinal spindle movement decreased by 7% (from 0.96 to 0.89) upon CENP-Ei treatment but equatorial and axial movements increased by 10% (from 0.23 to 0.32) and 2% (from 0.01 to 0.03), respectively (Fig. S13 c). In summary, although spindle movements are excessive and obvious following CENP-Ei treatment, the decomposition of spindle movements using SpinX reveals an increase in equatorial and axial movements and a reduction in longitudinal movements.

### MARK2 kinase inhibitor treatment promotes equatorial spindle movement

**Figure 6.**
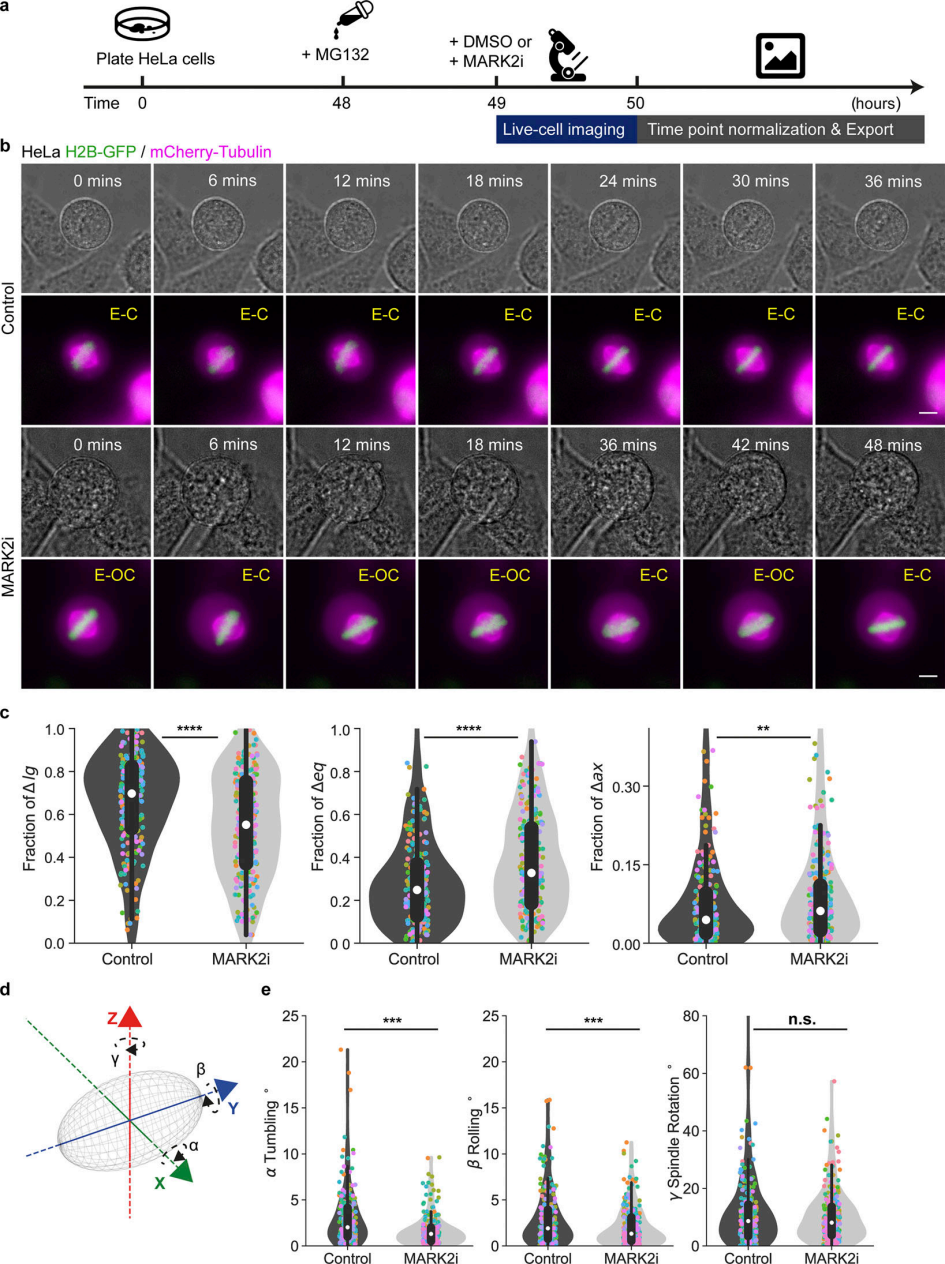
**MARK2i promotes equatorial movement of the mitotic spindle. (a)** Experimental regime. HeLa cells were exposed to MG132 (10 µM) 1 h before imaging. Either DMSO (control) or 5 µM MARK2 inhibitor (MARK2i) as indicated were added during imaging. **(b)** Representative brightfield images displaying cell cortex (gray) and maximum projection live-cell images of a HeLa cell expressing H2B-GFP (green) and mCherry-Tubulin (magenta). Equatorial-centered and off-centered spindles are marked (E-C) and (E-OC), respectively. Cells were imaged over 1 h with images taken every 3 min. **(c)** Violin plots show fractions of longitudinal (∆lg), equatorial (∆eq), and axial (∆ax) spindle displacement. Corresponding colored dots represent measurements from all time points of the same cell. The white marker within the box refers to the median, the shaded area refers to the estimated kernel probability density and the box indicates the interquartile range of the data. **(d)** Cartoon shows a 3D spindle (gray) with the corresponding rotation angles *α* (spindle tumbling), *β* (spindle rolling), and *γ* (spindle rotation) along its principal spindle axes *x,y,z*, respectively. **(e)** Violin plots show 3D angle distribution for *α* spindle tumbling, *β* rolling, and *γ* rotation. Corresponding colored dots represent measurements from all time points of the same cell. The white marker within the box refers to the median, the shaded area refers to the estimated kernel probability density, and the box indicates the interquartile range of the data. Statistical significance was determined by Mann–Whitney U test (in c and e) after a pre-analysis of the underlying distribution with a Shapiro–Wilk test. *N* = 11 control and *N* = 12 MARK2i cells across three separate experiments. Scale bars: 5 µm.

**Figure S14.**
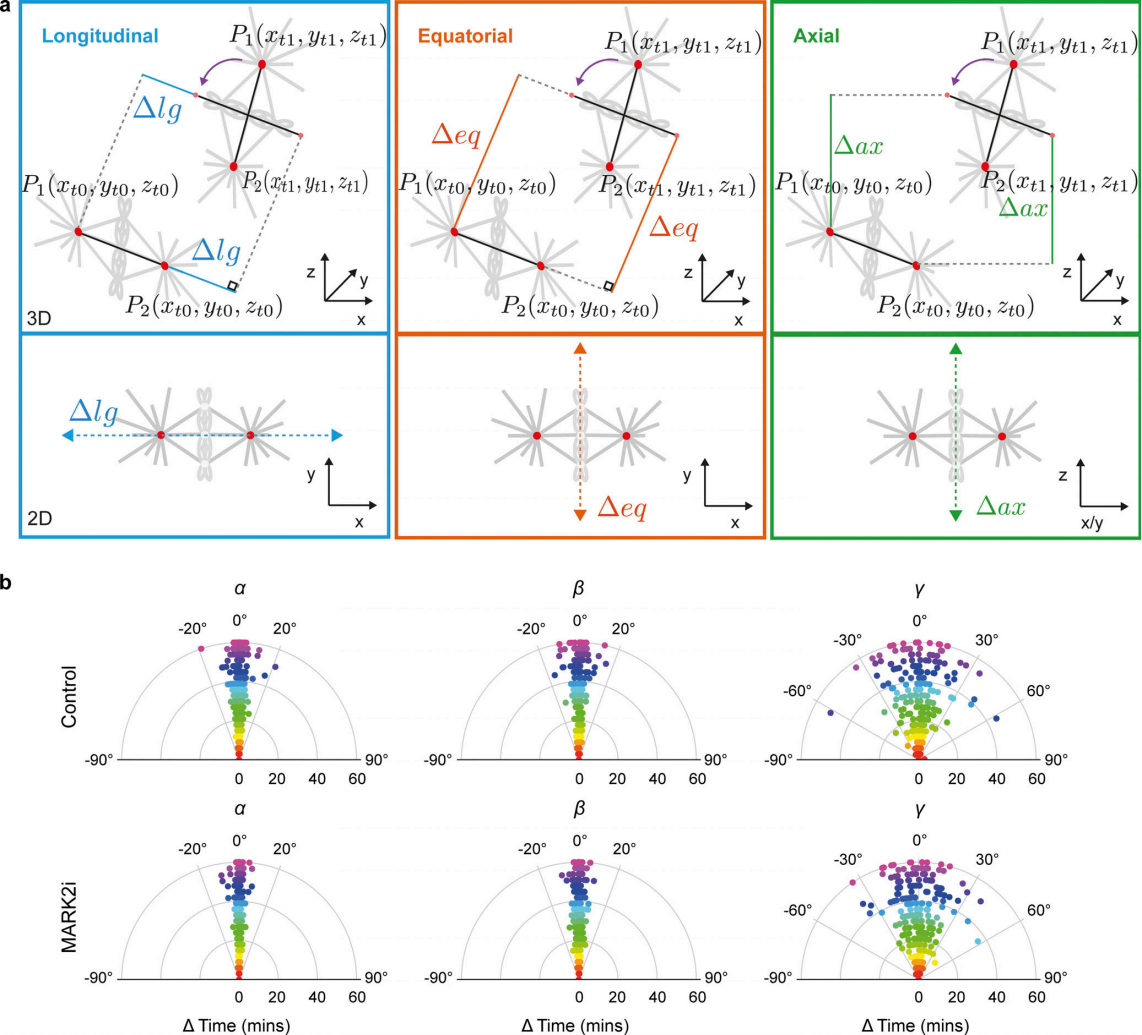
**MARK2i does not affect spindle rotation. (a)** Illustration shows decomposition of spindle movement (gray) from t_0_ to t_1_ in 3D. Red dots correspond to spindle poles *P*1 and *P*2. The spindle length axis is represented by the black line. Purple arc arrow indicates rotational movement of the spindle length axis at t_1_ when parallel to t_0_. Blue box: Blue lines cover the longitudinal movement ∆lg along the spindle length axis. Orange box: Orange lines cover the equatorial movement ∆eq along the spindle width axis which is perpendicular to the spindle length axis. Green box: Green lines cover the axial movement ∆ax along the spindle axial axis. The 2D representation (bottom row) illustrates the raw decomposed movement. **(b)** Polar scatter plot shows spindle rate of rotational movement (in degrees) through time. Measurements at each time point are highlighted with a unique color. *α*, *β*, and *γ* angles refer to spindle tumbling, rolling, and rotation respectively. Statistical significance was determined by Mann–Whitney *U* test after a pre-analysis of the underlying distribution with a Shapiro–Wilk test. *N* = 11 control and *N* = 12 MARK2i cells across three experiments.

To showcase the strength of an AI-based spindle tracking tool for high-throughput analysis of spindle movements in high-resolution time-lapse movies, we set out to quantify the consequence of exposing mitotic cells to an inhibitor of MARK2 (Microtubule Affinity Regulating Kinase 2, Par1 kinase family), implicated in centering spindles along the equatorial axis using protein depletion studies (Zulkipli et al., 2018). Whether loss of MARK2 activity can instantaneously disrupt spindle movements is not known. While a screen for drugs with therapeutic potential had identified an in vitro inhibitor of MARK2/Par1b activity (hereafter: MARK2i; Calbiochem 39621; Timm et al., 2011), whether this inhibitor can disrupt MARK2’s function in mitosis is not known. To address this, we collated 3D time-lapse movies of HeLa cells expressing H2B-GFP and mCherry-Tubulin (Fig. 6 a) exposed to MG132 (to enrich them in metaphase) in the presence or absence of MARK2i for up to 3 h. Visual inspection of time-lapse movies suggested that spindles of MARK2i-treated cells may be equatorially off-centered in some but not all timeframes (Fig. 6 b). To quantitatively assess changes in spindle movement in 3D, we used SpinX for tracking and decomposing spindle movements in longitudinal, equatorial, and axial orientations with respect to spindle length, width, and height axes, respectively (Fig. S14). By including the 3D cell cortex information, we could additionally account for variability in cell-to-cell differences, i.e., the available space for spindles to move and quantitatively compare across cells of variable sizes. In control metaphase cells (DMSO-treated cells)—as expected (Corrigan et al., 2013)—we observed a bias towards longitudinal movements of the spindle along the pole-to-pole axis and highly restricted equatorial movements (Fig. 6 c). However, in MARK2i-treated cells (*N* = 12), the fraction of longitudinal movement is significantly reduced, and the fraction of equatorial and axial movement are both increased, compared to control cells (*N* = 11; Fig. 6 c). The strong increase in equatorial movement following MARK2i treatment shows that the inhibition of MARK2 activity can promote equatorial movement, similar to MARK2 protein depletion (Zulkipli et al., 2018), revealing an immediate in vivo impact of the inhibitor and suggesting a close relationship between MARK2 activity and spindle movement regulation.

A defect in anaphase spindle orientation along the interphase long-axis after MARK2 depletion has been reported (Zulkipli et al., 2018), but changes in metaphase spindle orientation have not been previously quantified. We took advantage of SpinX’s reconstructed spindle principal axis and its corresponding centroid to compute 3D rotational angle changes in *α* spindle tumbling, *β* rolling, and *γ* rotation in metaphase spindles of control and MARK2i-treated cells (Fig. 6, d and e). We found that the extent of spindle rotation is not affected upon MARK2i (Fig. 6 e), but both the spindle tumbling and rolling movements are significantly reduced. To test if this reduction is time-dependent, we performed correlation analysis between angle changes and time (Fig. S14). Computed Pearson correlation coefficients *ρ* showed no linear correlation in both conditions in spindle tumbling (*ρ* = 0.101 and *ρ* = 0.060), spindle rolling (*ρ* = 0.102 and *ρ* = −0.090), and spindle rotation (*ρ* = 0.030 and *ρ* = 0.051). These findings reveal that MARK2i treatment alters spindle tumbling and rolling movements, but not rotational movements.

**Figure S15.**
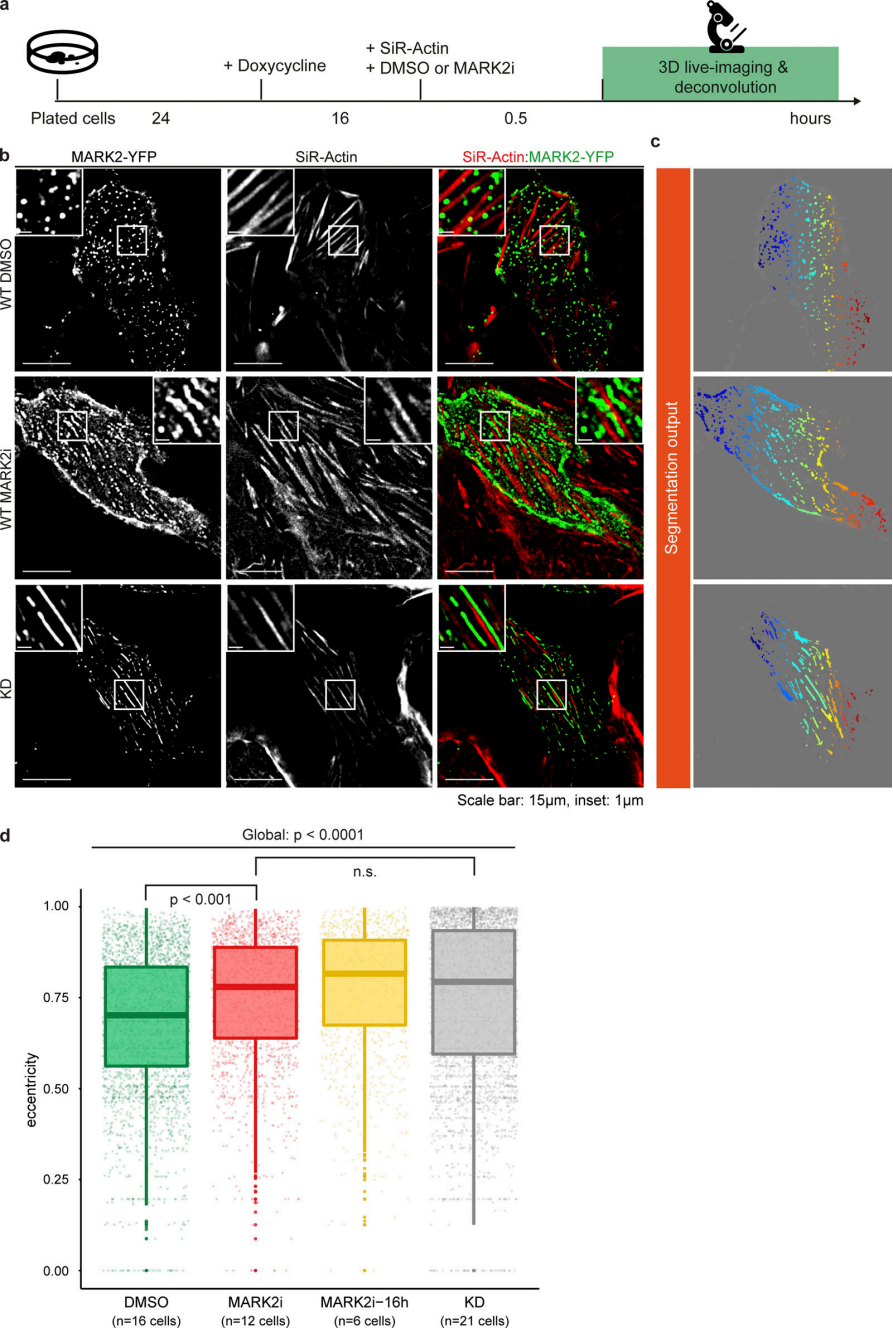
**MARK2-YFP localizes as punctuate-striation pattern upon MARK2 inhibition. (a)** Experimental regime and procedures. HeLa cells were exposed to Doxycycline 16 h before imaging. SiR-Actin (100 nM) and MARK2i (10 µM) or DMSO (solvent control) were added 30 min prior to imaging. **(b)** Deconvolved *z*-slice of 3D image stacks show MARK2-YFP (green) at the cell-substrate interface in WT treated with DMSO, MARK2i or in KD stained with SiR-Actin dye (red). White boxes show punctate foci pattern in WT (DMSO), punctate-striation pattern in WT (MARK2i) and long striation in KD. **(c)** Segmentation results of MARK2-YFP signal for quantification after manually outlining interphase cell boundaries. **(d)** The boxplots show eccentricity measurements for DMSO, MARK2i, MARK2i prolonged treatment (16 h) and KD. 0 represents a perfect circle and 1 a perfect line. For global group comparison, a generalized linear model (GLM) was fitted (non-normality was determined with Shapiro-Wilk test) followed by a post-hoc analysis for pair-wise comparison (Dunn’s post-hoc test) after Multiple-Comparison Kruskal–Wallis (MCKW) at a significant level of P < 0.01. *n* refers to the number of cells obtained from three experiments. Scale bars: 15 µm; 1 µm for inset.

As SpinX-based spindle tracking helped uncover the immediate in vivo impact of the MARK2 inhibitor in mitotic cells, we used the same concentration of MARK2i that altered spindle movements (Fig. 6) to test if transient exposure to the inhibitor is sufficient to alter MARK2 localization during interphase. Interphase localization of MARK2 is dependent on its activity: while MARK2 WT localizes as puncta throughout the interphase cell–substrate interface, the kinase-dead (KD) mutant localizes as long striations parallel to actin fibers (Hart et al., 2019). Following a brief 30-min exposure to MARK2i, MARK2-YFP localized as long striations parallel to actin fibers near the cell substrate (Fig. S15, a and b). In contrast to the prominent punctate-foci distribution of MARK2-YFP in control cells treated with DMSO, MARK2i-treated cells showed fewer punctate-foci but a higher number of long striations along the actin stress fibers (Fig. S15 b), indicating a change in MARK2 localization following MARK2i treatment. Segmentation and quantification of eccentricity of MARK2-YFP foci (Fig. S15 c) confirmed that the localization of MARK2-YFP was significantly altered upon MARK2 inhibition, representing eccentricity values similar to the distribution of foci in MARK2-KD expressing cells (Fig. S15 d). Prolonged MARK2 inhibitor treatment, by exposing cells for a longer period (16 h), did not significantly change the localization pattern compared to a shorter period of drug treatment (Fig. S15 d), demonstrating the in vivo use of MARK2 inhibitor to acutely block MARK2 function during both interphase and mitosis. Thus, SpinX enabled precise tracking of 3D spindle movements following inhibitor treatment(s), showcasing the robustness of DL-based quantitative analysis of discontinuous time-lapse movies.

## Discussion

We showed that an AI-based image analysis framework supported by 3D modeling can harness dynamic information in time-lapse microscopy movies in a quantitative manner. By bringing together large-scale time-lapse movie datasets and the SpinX computational framework, we can now precisely track spindle movements, in 3D, using diverse spindle protein markers allowing the possibility of a variety of high-throughput drug development or drug target screens. Using manual and automated benchmarking tools, we establish that SpinX can reliably (i) detect and segment the spindle and the cell membrane, (ii) transform 2.5D data to true 3D through ellipsoid reconstruction, and (iii) track spindle movement relative to cell size through 3D mathematical modeling. We compared our methods to existing ones for segmentation, Spindle3D and Cellpose (Kletter et al., 2022; Stringer et al., 2021), and highlight the strengths of SpinX in accurate segmentation of spindles and precise tracking of spindle movements in 3D. The methods we present here can be of general use beyond spindle tracking, for example, 3D reconstruction for fluorescent images by utilizing properties of the PSF, ray-tracing principle to model 3D movements relative to different subcellular structures, a six-point 3D tracking algorithm for capturing translational and rotational movements of structures, and an expert error classification system to support model evaluation and refinement. Last, we showcased SpinX’s potential in supporting preclinical cell biology research and drug development studies by taking advantage of the complexity of mitotic spindle movements that we accurately measure in cells treated with chemical inhibitors of CENP-E kinesin or MARK2 kinase.

**Figure 7.**
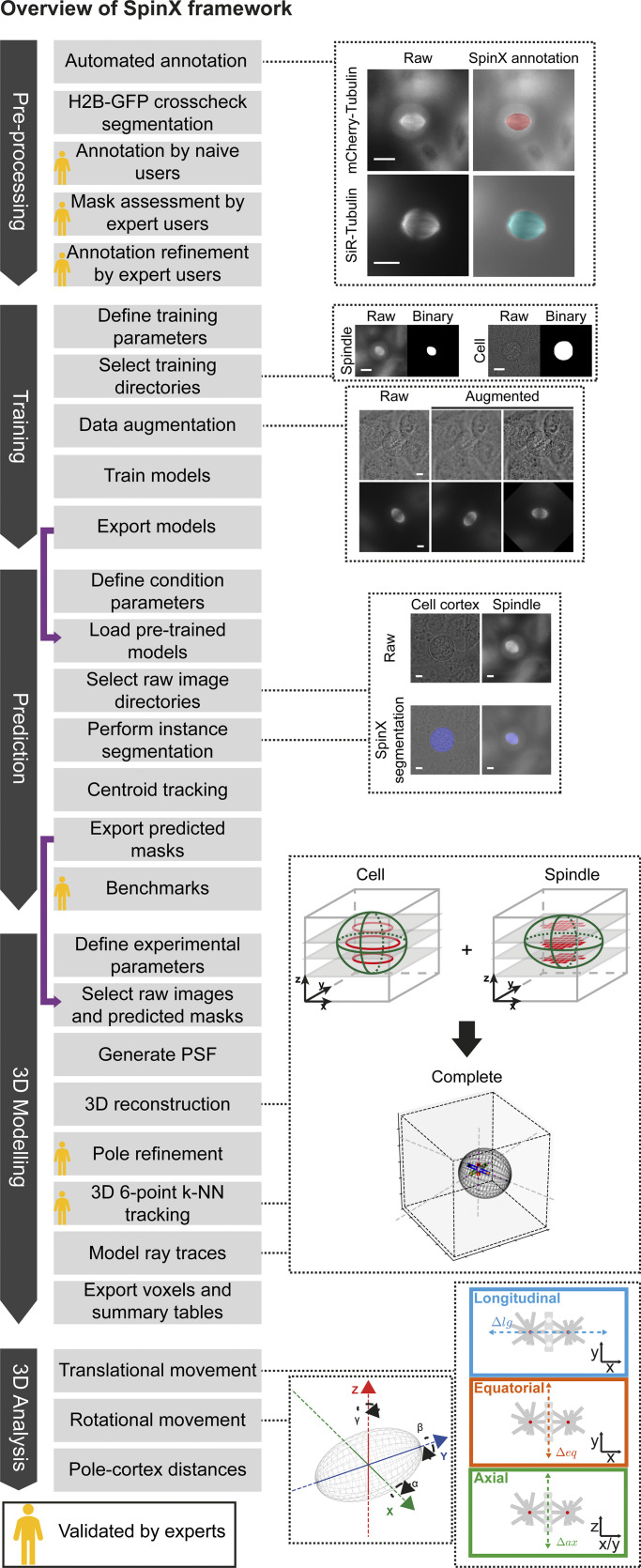
**SpinX’s comprehensive framework.** Diagram shows the complete framework of SpinX including modules for generating annotations (pre-processing), training, prediction, 3D modeling and 3D analysis (dark gray arrows). Each module has a series of automated and manual steps (light gray boxes), with purple arrows indicating how data is passed between modules. Representative raw images and their corresponding automatically annotated spindle images, along with raw and binary spindle and cell cortex images belonging to the training dataset are shown. 3D reconstructions of the cell cortex and mitotic spindle enable the extraction of translational and rotational spindle movements, and pole-to-cortex distances. Image annotation, predicted masks enabling temporal and spatial links between images, and 3D modeling of pole position and tracking are all validated by experts (gold icons). Scale bars: 10 µm.

One of the major hurdles in DL-based tool development is the lack of large volumes of high-resolution time-lapse datasets that are essential for feature-rich analysis of subcellular structures. However, the lack of sophisticated image analysis tools discourages the generation of such large-scale high-resolution datasets. Here we break this conflicting scenario by generating both time-lapse movie datasets and analysis tools for measuring and characterizing spindle size, position, and movements in 3D. Thus, SpinX provides a complete framework including modules for annotation, training, modeling, tracking, and analysis, and the possibility of validating predictions at multiple steps of the process (Fig. 7), enabling robust 3D tracking of spindle movements relative to the cell cortex.

SpinX’s contribution to the live-cell microscopy field is twofold: extending the Mask R-CNN network to perform predictions on 3D time-lapse movie datasets and building a high-resolution fully annotated dataset of images of fluorescently labeled spindles and label-free cells. The Mask R-CNN-based architecture allowed us to harness the network’s flexibility in handling images of arbitrary size, and supported instance segmentation of multiple classes—a crucial feature for cell segmentation to separate overlapping cells, while also classifying them into distinct phenotypes and providing unique IDs. The evaluation of the neural network model using a detailed error classification system helped assess the strengths and limitations of SpinX. For example, errors in cell cortex prediction were mostly categorized as “undersegmentation,” which were mathematically compensated by the MVEE during modeling. Our findings highlight the importance of annotation quality, especially for studies where precise measurements of object boundaries are important for accurate 3D modeling and object tracking through time. Benchmarking studies with expert and beginner users confirmed the benefits of Mask R-CNN, including the generalization capacity of SpinX to detect spindle markers, cell types and microscopes beyond the ones used to train the model. In cases where further improvement in segmentation may be required (e.g., bovine two-cell embryos), further retraining is possible, for example, using transfer learning, which has been shown to be a more effective approach than retraining from scratch (Vasconcelos et al., 2022), to take full advantage of the 3D modeling and tracking modules of SpinX.

Unlike manual analysis of spindle movements or previous spindle tracking efforts (Corrigan et al., 2013; Larson and Bement 2017), automated analysis using SpinX can capture translational and rotational movements of subcellular structures using the six-point 3D tracking algorithm, and measure 3D spindle movements relative to the cell cortex using principles from ray tracing methods. This allowed the first careful assessment of the impact of the inhibitors on metaphase spindle movements in 3D. MARK2 inhibitor treatment does not affect spindle rotation per se, but affects spindle rolling and tumbling by altering the equatorial positioning of spindles. These findings are consistent with equatorial positioning defects, previously reported through manual analysis of MARK2 depleted cells (Zulkipli et al., 2018). Similarly, SpinX analysis shows that CENP-E inhibitor treatment that promotes excessive spindle movements increases axial or equatorial movements more extensively compared to longitudinal movements. In summary, we expected this advance in measuring spindle movements through SpinX to help dissect molecular regulators responsible for precisely guiding the spindle’s movement to its final position.

The SpinX 3D-modeling module used for precise tracking is limited to cases where the mitotic spindle can be extrapolated to an ellipsoid. However, it can be used for a variety of cells including mouse ESCs, kidney epithelial cells and bovine oocytes. This extrapolation is expected to work in most mammalian cells as spindle width is a good predictor of spindle size (Kletter et al., 2022). In unusual scenarios of longer or wider spindles, it’s possible to fine-tune the eccentricity of the ellipsoid using spindle width as a parameter.

As the mitotic spindle movements are highly sensitive to changes in the cell’s cytoskeleton, membrane compartment, and chromosome position, SpinX-supported spindle movement analysis is expected to help accelerate and advance automated screening of drug targets and chemical compounds that act on cytoskeletal and membrane compartments. In addition, SpinX developed for single-cell studies, based on Mask R-CNN, can be readily generalized to multi-cell images and also multi-content images to allow the simultaneous tracking of more than one subcellular structure.

## Materials and methods

The SpinX framework was developed in Python 3, using Numpy, Scipy, Tensorflow, Keras, Scikit, Pandas, and opencv. For the interactive interface, Tkinter was used. Figures were generated using Matplotlib, Seaborn, and Jupyter Notebook.

### Data generation

#### Cell line and culture conditions

HeLa cell lines (ATCC CCL2) used were cultured in Dulbecco’s Modified Eagle’s Medium (DMEM) supplemented with 10% fetal bovine serum (FBS; 10270106; Thermo Fisher Scientific), 1% Penicillin/Streptomycin (15140122; Thermo Fisher Scientific), and 0.1% Amphotericin B (Fungizone; 11510496; Thermo Fisher Scientific). Cell lines were cultured as a monolayer at 37°C and 5% CO_2_. HeLa H2B-GFP, mCherry-Tubulin cell line was generated by transfecting mCherry-Tubulin expressing eukaryotic plasmid vector into HeLa H2B-GFP cells (Draviam et al., 2006). The HeLa mKate2-EB3 cell line was generated by transfecting a pmKate2-EB3 plasmid vector (#FP316; Evrogen) into HeLa cells. Plasmid transfection was carried out using DharmaFECT duo (T-2010; Dharmacon). The HeLa H2B-GFP, SiR-Tubulin cell line was generated by adding SiR-Tubulin dye, a paclitaxel-based fluorescent compound (Lukinavičius et al., 2014; Spirochrome SC002; 100 nM) just 1 h before imaging. The HeLa FRT/TO cell line expressing siRNA-resistant MARK2-YFP-WT or KD mutant was generated by transfecting a Tet-inducible expression vector encoding siRNA-resistant MARK2-YFP-WT or KD, followed by colony picking (Zulkipli et al., 2018). Vectors bearing point mutants of MARK2 were generated by polymerase chain reaction–based point mutagenesis and confirmed by DNA sequencing (Hart et al., 2019). Fluorescent cells were enriched using a BD FACSAria III Cell Sorter for fluorescence-activated cell sorting (FACS).

#### Live-cell microscopy

Live-cell imaging experiments were performed using cover glass chambered dishes (155383PK; Thermo Fisher Scientific). MG132 (1748; Tocris Biosciences; 10 µM) was added 1 h before imaging to synchronize cells at metaphase (Shrestha et al., 2017; Iorio et al., 2015). During imaging, cells were incubated in Leibovitz’s L15 medium (11415064; Thermo Fisher Scientific). For MARK2 studies, MARK/Par-1 activity inhibitor (Timm et al., 2011; MARK2i; 39621; Calbiochem; 5 or 10 µM) was added prior to imaging. For CENP-E studies, CENP-Ei (GSK-923295; MedChemExpress; 30 nM) was added prior to imaging.

For HeLa FRT/TO MARK2-YFP (WT and KD) experiments, Doxycycline (10224633; Thermo Fisher Scientific; 200 ng/ml) was added 16 h prior to imaging (Zulkipli et al., 2018). SiR-Actin dye (Lukinavičius et al., 2014; SC001; Spirochrome; 100 nM) was added 30 min before imaging. All imaging sessions were conducted in a chamber at 37°C.

Widefield images were acquired with an Applied Precision Deltavision Core deconvolution microscope equipped with a dual camera system composed of a CoolSNAP and Cascade2 Camera (Photometrics) under EM mode. For live-cell studies, images were taken every 3 min (21 timepoints—total time 60 min) with optimized exposure times ranging from 0.1 to 0.2 s depending on the imaging channel. For each experiment, at least three *z*-sections (2 µm gap) were acquired using an oil-based 60X NA 1.42 objective or 100X NA 1.40 objective. High-resolution imaging datasets have pixel sizes ranging between 0.04144 and 0.06887 µm. Time-point equalization, deconvolution, and data export (Tiff-format) were performed in softWoRx 6.5.2.

Confocal images were acquired using a Leica Stellaris 8 confocal microscope with an oil-based 63X NA 1.40 objective. Each movie consisted of at least four *z*-sections (max 46) taken with 0.2–0.5 µm gaps. All movies underwent adaptive deconvolution (Lightning mode). Before processing through SpinX, movies were converted to 8-bit and padded to 1,024 × 1,024 dimensions using our padding algorithm (described below).

#### Image datasets

Our image pools include 13,230 cell membrane and 15,120 spindle images of HeLa cervical epithelial cells. Cell membrane images were pooled from 188 3D high-resolution live-cell movies, while the spindle images were pooled from 217 3D high-resolution live-cell movies, both across 26 experiments. A uniform random generator was used to randomly select 2,180 cell membrane and 2,320 spindle images to build the training, validation, and testing datasets (Fig. S1). For SpinX’s final cell membrane model (i.e., SpinX-optimized) the training dataset consisted of 1,300 images (Tables S2, S3, and S4; and Figs. S5 and S6), the validation dataset consisted of 250 images (Tables S4, and Figs. S5 and S6), and the testing dataset consisted of 630 images (Fig. 2). For SpinX’s final spindle model (i.e., SpinX-optimized) our training dataset consisted of 1,390 images (Tables S2, S3, and S4; and Figs. S5 and S6), the validation dataset consisted of 300 images (Tables S3 and S4; and Figs. S5 and S6), and the testing dataset consisted of 630 images (Fig. 2). For testing the generalization extent of SpinX high-resolution 3D live-cell time-lapse datasets of HeLa cells expressing mRFP-EB3 (1,540 images from 5 cells), YFP-Astrin (330 images from 10 cells) and mKate2-EB3 (1,920 images from 5 cells) were used. In addition, datasets provided by Spindle3D (Kletter et al., 2022) were used, including bovine one-cell embryos (30 images from 10 embryos), bovine two-cell embryos (30 images from 10 embryos), HEK293 cells (130 images from 10 cells), and mESC cells (50 images from 10 cells), wherein images displaying spindles with a visible midzone were chosen.

#### Annotation

Manual annotations required for training and evaluation (i.e., ground-truth masks) were performed with VGG Image Annotator (VIA) tool (Dutta and Zisserman 2019). Any mis-segmented images from SpinX’s AI output were also manually corrected through VIA before 3D reconstruction and modeling. Automated annotations were generated through chromosome and spindle segmentation pipelines combining different conventional image processing techniques. The chromosome segmentation pipeline used for segmenting H2B-GFP labeled chromosomes includes: (1) a median filter for noise reduction (Huang et al., 1979); (2) Otsu’s method for iterative two-class thresholding (by minimizing the weighted within-class variance), thereby globally reducing a grayscale image to a binary image (Otsu 1979); (3) a connectivity matrix making up an 8-connected neighborhood used for clearing any pixels found at the image border; and (4) contour smoothing with the Savitzky-Golay signal processing filter (Orfanidis 1996; Fig. S2 a). The spindle segmentation pipeline used for segmenting SiR-Tubulin labeled spindles includes: (1) median filtering of the size [20, 20] for improving signal-to-noise ratio; (2) an adaptive threshold for estimating the average background illumination intensity; (3) binarization along with morphological dilation and erosion for removing artifacts; (4) calculating the convex hull of the segmented spindle halves to allow joining; (5) contour smoothing with the Savitzky-Golay signal processing filter (Orfanidis 1996); and (6) fitting an ellipse to obtain spindle properties (Fig. S2 b). The spindle segmentation pipeline used for segmenting mCherry-Tubulin labeled spindles includes: (1) a Gaussian filter for noise reduction; (2) calculation of the image gradient; (3) an automated snake i.e., active contour model (Kass et al., 1988), that uses boundary information from the already segmented chromosomes as initial coordinate points; and (4) inversing the snake, thereby propagating from the center of the spindle towards the outer boundary contour to avoid any cytoplasmic noise (Fig. S2 c). All automatically generated annotations were manually assessed and corrected if needed using VIA tool (Dutta and Zisserman 2019).

### Deep neural network

#### ResNet CNN for DL model of spindle and cortex

The ResNet CNN computes full-image feature maps with an increased depth of 101 layers, therefore achieving an elevated semantic value, despite the progressive loss in spatial resolution. The final feature map generated is fed into the RPN, leading to thousands of propositions of where the object of interest is most likely located, termed as regions of interest (ROIs). The presence of an RPN in the architecture allows the detection of individual cells within densely populated images, while also enabling the tracking of the same object across time through the bounding boxes generated. The RPN defines several sets of bounding boxes by a sliding window approach which is based on a computed IoU metric (Ren et al., 2017). The sets of bounding boxes then undergo binary classification and regression in a parallel manner, followed by non-maximum suppression for selecting the most accurate non-overlapping bounding boxes (Girshick et al., 2015; He et al., 2018). The resulting bounding boxes (i.e., anchor boxes) indicating the same ROI are aligned with each other through bilinear interpolation—also known as the ROIAlign layer, which improves pixel accuracy through the refinement of pooling operations (i.e., object extrema; He et al., 2018). Subsequently, the FCN allows the simultaneous prediction of the corresponding class and bounding box for each ROI from the detection network, and the generation of the mask within each ROI from the segmentation network.

#### Data augmentation

For both brightfield and fluorescent images used for training the cell cortex and spindle models respectively, augmentation was carried out on every epoch. Augmentation techniques used included image blurring through Gaussian filtering, contrast normalization, translation, rescaling between 80 and 120%, rotating up to 180° or shearing by -8–8° (Fig. S4). Images also underwent flipping, element-wise addition, simple pixel value addition and multiplication, random pixel dropout of up to 10%, gamma adjustment, and cropping (Fig. S4). For the cell cortex models priority was given to translation, rescaling and shearing to address the natural variation in cell size and shape; whereas for the spindle models priority was given to rotation and flipping to capture the variety in spindle dynamics (Fig. S4).

#### Training

For training our Mask R-CNN models, we used strategies from Abdulla (2017). The networks were trained for at least 200 epochs (base models) or 500 epochs (optimized models) with stochastic gradient descent at a learning rate of 0.001, a momentum of 0.9, batch size of one image and a weight decay of 0.001 (Table S3). The number of anchors for RPN was set to 512. The detection threshold was set at 90%. Models were initiated with COCO pre-trained weights (Lin et al., 2015). The best models were selected based on the lowest loss value in the training and validation datasets. To train U-Net, we used a learning rate of 0.00001 with a batch size of 4 and trained for 500 epochs.

#### Metrics

IoU scores were calculated by quantifying the matching between predictions proposed by DL models and their corresponding ground-truth masks. Average Precision (AP) scores to assess class assignment are defined as $AP=\sum_{n}\left(R_{n}-R_{n-1}\right)P_{n}$, where *P*_*n*_ and *R*_*n*_ are the precision and recall at the *n*th threshold (Table S3). Loss functions were determined as described in He et al. (2018) (Fig. S6 and Table S3).

### Padding

Padding of the YFP-Astrin and mKate2-EB3 datasets were performed through an algorithm that extracted small-sized patches of the input image based on their sum of pixel intensity values. The patches exhibiting the lowest sum of intensity values were then used to pad the input image to a desired size. Therefore, the low-intensity small-sized patches emulated and propagated the “background” of the input image. This transformed the Astrin-YFP dataset to 512 × 512 pixel images and the mKate2-EB3 dataset to 1,024 × 1,024 pixel images, subsequently enabling SpinX’s AI module to segment spindles.

### Statistical analysis

Statistical tests were performed in Python 3 (using Scipy package) and R-Studio (using R 3.6). Statistical tests were performed on a significance level of P ≤ 0*.*01 or P ≤ 0*.*05. For P values, the following convention holds: not significant (n.s.) with P *>* 0*.*05, (*) with P ≤ 0*.*05, (**) with P ≤ 0*.*01, (***) with P ≤ 0*.*001 and (****) with P ≤ 0*.*0001.

### PSF to estimate spindle pixels in z

PSF was simulated with the Gibson-Lanni model (Gibson and Lanni 1992) that accounts for different imaging conditions (Table S6). To translate the empirical measurements of the PSF to a mathematical function, the intensity values on the *x,y* and *z*-sections were fitted. Given a 3D image stack of a fluorescence bead where *x* and *y* are intensity values, (*x*_*c*_*,y*_*c*_) is the centroid coordinates of the brightest spot across the *z*-stack, *h* is the height of the Gaussian and *σ*_*x*_ and *σ*_*y*_ are variances in the *x* and *y* directions where $\left(\sigma_{x}\neq \sigma_{y}\right)$. Then, the 2D Gaussian with *k* = 2 is the product function derived from a multivariate Gaussian X_∈(*x,y*)_ ∼ *N*_*k*=2_(*µ,*Σ) where $\Sigma \;=\;\left[\begin{array}{c} \sigma_{x}^{2}\\ 0 \end{array}\begin{array}{c} 0\\ \sigma_{y}^{2} \end{array}\right]$ with det(Σ) = *σ*_*x*_^2^*σ*_*y*_^2^:

$$fX\left(x,y\right)=\frac{A}{\sqrt{{\left(2\pi \right)}^{k}|\Sigma |}}\exp\left\{-\frac{1}{2}{\left(x-\mu \right)}^{T}\Sigma^{-1}\left(x-\mu \right)\right\}\text{,}$$

$$=\frac{A}{\sqrt{{\left(2\pi \right)}^{2}\sigma_{x}^{2}\sigma_{y}^{2}}}\exp\left\{-\;\frac{1}{2}\left[\begin{array}{cc} x-x_{c} & y-y_{c} \end{array}\right]\left[\begin{array}{cc} \frac{1}{\sigma_{x}^{2}} & 0\\ 0 & \frac{1}{\sigma_{x}^{2}} \end{array}\right]\left[\begin{array}{c} x_{1}-x_{c}\\ y_{1}-y_{c} \end{array}\right]\right\}\text{,}$$

$$=\frac{A}{2\pi \sigma_{x}\sigma_{y}}\exp\left\{-\;\frac{{\left(x-x_{c}\right)}^{2}}{2\sigma_{x}^{2}}\;-\;\frac{{\left(y-y_{c}\right)}^{2}}{2\sigma_{y}^{2}}\right\}\text{.}$$ (1)

To fit *z* data points of intensity values of the fluorescence bead along the *z*-slices, Eq. 1 can be simplified to a to 1D Gaussian with:

$$f(z)=\frac{A}{2\pi \sigma_{z}^{2}}\exp\left\{-\frac{{\left(z-z_{c}\right)}^{2}}{2\sigma_{z}^{2}}\right\}\text{.}$$ (2)

Then, the function that relates the intensity values to the estimated *z*-coordinate $\hat{z}$ with respect to the reference intensity profile *r*_*int*_ is

$$I=\exp\left[-1{\left(\frac{z-z_{c}}{\sigma }\right)}^{2}\right]\text{.}$$ (3)

For each pixel, we, therefore, applied the following equation to estimate its $\hat{z}$-coordinate:

$$\hat{z}=\sigma \sqrt{-1*log\left(I\right)+z_{c}}\;\text{,}$$ (4)

where *z*_*c*_ and *σ* denotes the mean and variance of the Gaussian. To then 3D reconstruct the spindle, we use its assigned prediction mask generated by the SpinX AI module and burn it on the initial raw fluorescent image. This step isolates neighboring signal noise and retains only the pixels belonging to the spindle. Then, we filtered the predicted mask by keeping the top 30% of pixels with the highest intensity, thus reducing the number of data points while maintaining the shape of the spindle.

### Ray traces to determine pole-to-cortex distance

Given a line in a three dimensional space which is defined by two points *P*_1_(*x*_1_*,y*_1_*,z*_1_) and *P*_2_(*x*_2_*,y*_2_*,z*_2_), the parametric line for points of intersect can be described by

$$P=P_{1}+t\left(P_{2}-P_{1}\right)\text{,}$$ (5)

where each coordinate of P can be written as

$$x=x_{1}+t\left(x_{2}-x_{1}\right)$$

$$y=y_{1}+t\left(y_{2}-y_{1}\right)$$

$$z=z_{1}+t\left(z_{2}-z_{1}\right)\text{.}$$ (6)

An ellipsoid translated to its center at *P*_3_(*x*_3_*,y*_3_*,z*_3_) can be described by

$$\frac{{\left(x-x_{3}\right)}^{2}}{a^{2}}+\frac{{\left(y-y_{3}\right)}^{2}}{b^{2}}+\frac{{\left(z-z_{3}\right)}^{2}}{c^{2}}=1\text{.}$$ (7)

The intersection points *P* of the parametric Eq. 5 satisfy the substituted Eq. 6 in Eq. 7:

$$\frac{{\left[\left(x_{1}-x_{3}\right)+t\left(x_{2}-x_{1}\right)\right]}^{2}}{a^{2}}+\frac{{\left[\left(y_{1}-y_{3}\right)+t\left(y_{2}-y_{1}\right)\right]}^{2}}{b^{2}}+\frac{{\left[\left(z_{1}-z_{3}\right)+t\left(z_{2}-z_{1}\right)\right]}^{2}}{c^{2}}=1\text{.}$$ (8)

Solving the square values of the parenthesis yields:

$$\frac{{\left(x_{2}-x_{1}\right)}^{2}t^{2}-2t\left(x_{2}-x_{1}\right)\left(x_{1}-x_{3}\right)+{\left(x_{3}-x_{1}\right)}^{2}}{a^{2}}+$$ (9)

$$\frac{{\left(y_{2}-y_{1}\right)}^{2}t^{2}-2t\left(y_{2}-y_{1}\right)\left(y_{3}-y_{1}\right)+{\left(y_{1}-y_{3}\right)}^{2}}{b^{2}}+$$ (10)

$$\frac{{\left(z_{2}-z_{1}\right)}^{2}t^{2}-2t\left(z_{2}-z_{1}\right)\left(z_{1}-z_{3}\right)+{\left(z_{3}-z_{1}\right)}^{2}}{c^{2}}-1=0\text{.}$$ (11)

Arranging the expression received as powers of *t* yields:

$$\left[\frac{{\left(x_{2}-x_{1}\right)}^{2}}{a^{2}}+\frac{{\left(y_{2}-y_{1}\right)}^{2}}{b^{2}}+\frac{{\left(z_{2}-z_{1}\right)}^{2}}{c^{2}}\right]t^{2}+\left[\frac{2\left(x_{2}-x_{1}\right)\left(x_{1}-x_{3}\right)}{a^{2}}+\frac{2\left(y_{2}-y_{1}\right)\left(y_{1}-y_{3}\right)}{b^{2}}+\frac{2\left(z_{2}-z_{1}\right)\left(z_{1}-z_{3}\right)}{c^{2}}\right]t$$

$$+\left[\frac{{\left(x_{1}-x_{3}\right)}^{2}}{a^{2}}+\frac{{\left(y_{1}-y_{3}\right)}^{2}}{b^{2}}+\frac{{\left(z_{1}-z_{3}\right)}^{2}}{c^{2}}-1\right]=0\text{.}$$ (12)

Substituting the equation of the line into the ellipsoid form gives a quadratic equation of the form:

$$\chi t^{2}+\gamma t+\zeta =0\text{,}$$ (13)

where:

$$\chi =\frac{{\left(x_{2}-x_{1}\right)}^{2}}{a^{2}}+\frac{{\left(y_{2}-y_{1}\right)}^{2}}{b^{2}}+\frac{{\left(z_{2}-z_{1}\right)}^{2}}{c^{2}}\text{,}$$ (14)

$$\gamma =\frac{2\left(x_{2}-x_{1}\right)\left(x_{1}-x_{3}\right)}{a^{2}}+\frac{2\left(y_{2}-y_{1}\right)\left(y_{1}-y_{3}\right)}{b^{2}}+\frac{2\left(z_{2}-z_{1}\right)\left(z_{1}-z_{3}\right)}{c^{2}}\text{,}$$ (15)

$$\zeta =\frac{{\left(x_{1}-x_{3}\right)}^{2}}{a^{2}}+\frac{{\left(y_{1}-y_{3}\right)}^{2}}{b^{2}}+\frac{{\left(z_{1}-z_{3}\right)}^{2}}{c^{2}}-1.$$ (16)

The solution for *t* is then:

$$t=\frac{-\gamma \pm \sqrt{\gamma^{2}-4x\zeta }}{2\chi }\text{,}$$ (17)

where

$$t<0.0\left(no\;intersections\right)\text{,}$$ (18)

$$t=0.0\left(one\;intersection\right)\text{,}$$ (19)

$$t>0.0\left(two\;intersections\right)\text{.}$$ (20)

Substituting *t* in Eq. 5 yields the intersection points P where the spindle axis collides with the cell cortex. By applying the analytical solution, the precise intersection points can be derived at a lower computational cost (Fig. S11). Eq. 4 was derived by (i) fitting a 2D Gaussian function along the *xy* coordinate of the PSF and (ii) a 1D Gaussian fit at the centroid of the PSF along the *z*-slices.

### SpinX pole location refinement

The spindle pole refinement algorithm takes initial (x,y,z) spindle pole predictions as an input. The spindle boundary coordinates are obtained by taking the maximum projection of the spindle mask. Then, 3D coordinates are extracted along the spindle length axis (pole-to-pole axis) to obtain the corresponding pixel values. The true spindle pole is defined as the first and last occurrence of positive values in the resulting 1D array. Finally, the new position of spindle poles was updated across all data frames for further calculations (Table 1). Manual analysis used for evaluating the performance of SpinX’s pole position recording was performed on Fiji (ImageJ) software (Schindelin et al., 2012).

**Table 1.** SpinX pole refinement algorithm to compensate overestimation of spindle length axis

Steps	Input	Output
1	Perform MVEE as usual based on spindle signal to predict pole position	First estimation of (*x,y,z*) for Poles 1 and 2
2	Create max projection of the spindle mask to identify spindle boundary	Max projection of mask
3	Extract 3D coordinates along the spindle length axis (pole-to-pole axis) and obtain the corresponding pixel values (conversion from float to integer results in rounding bias)	1-D array with pixel values obtained from the max projected mask
4	Identify the first and last occurrence of the array which corresponds to the poles	Refined (*x,y,z*) for Poles 1 and 2
5	Re-calculate the centroid and radius of the refined poles and use them as input to re-apply ellipsoid fitting to update data frame and generated plots (increase computational run time marginally)	Updated data frame

### SpinX pole identity assignment

We implemented a six-point tracking algorithm based on *k*-Nearest Neighbor algorithm (*k*-NN). The six points represent the end points of the three principal axes of the ellipsoid which corresponds to spindle height, width, and length axis and works as follows:

Given (*x,y,z*) coordinates of *i* = 2 poles at *j* = 2 consecutive time points P_1(*t,t*−1)_ and P_2(*t,t*−1)_, the pairwise distance between poles can be described by the distance matrix

$$D\left(i,j\right)=d\left(P_{i,t},P_{j,t-1}\right)\text{,}$$ (21)

where d is the 3D Euclidean distance between two consecutive points

$$if\;min\left(d\left(P1_{t},P1_{t-1}\right)\right),\;no\;correction$$

$$if\;\min\left(d\left(P1_{t},P2_{t-1}\right)\right),\;correction\;applied$$

$$if\;\min\left(d\left(P2_{t},P1_{t-1}\right)\right),\;correction\;applied$$ (22)

$$if\;\min\left(d\left(P2_{t},P2_{t-1}\right)\right),\;no\;correction$$

The condition for a correct assignment of pole 1 at *t* is when its distance is smallest at *t* −1 and largest to pole 2 at *t* −1. Based on this condition, SpinX performs correction for individual poles whenever they were falsely assigned (e.g., pole 1 at *t* −1 is closest to pole 2 at *t*). Once corrected, SpinX updates the correction throughout the data frame. To test how frequently corrections have to be applied, we analyzed 10 randomly selected time-lapse movies. According to Table S5, correction with *k*-NN was required for around half of the time for spindle width and length axes and one third for spindle height axis, respectively (Fig. 4 e).

### Computing 3D rotational movement with Euler’s angle

The extent of spindle rotation is defined by the rotation matrix (R_3×3_) which is the product of successive rotation about the *z*, *y* and *x* axes such as:

$$R=R_{z}\left(\gamma \right)R_{y}\left(\beta \right)R_{x}\left(\alpha \right)$$ (23)

with

$$R_{x}\left(a\right)=\left[\begin{array}{ccc} 1 & 0 & 0\\ 0 & cos\left(\alpha \right) & sin\left(\alpha \right)\\ 0 & -sin\left(a\right) & cos\left(a\right) \end{array}\right]\text{,}$$ (24)

$$R_{y}\left(\beta \right)=\left[\begin{array}{ccc} cos\left(\beta \right) & 1 & -sin\left(\beta \right)\\ 0 & 1 & 0\\ sin\left(\beta \right) & 0 & cos\left(\beta \right) \end{array}\right]\text{.}$$ (25)

$$R_{z}\left(\gamma \right)=\left[\begin{array}{ccc} cos\left(\gamma \right) & sin\left(\gamma \right) & 0\\ -sin\left(\gamma \right) & cos\left(\gamma \right) & 0\\ 0 & 0 & 1 \end{array}\right]$$ (26)

and satisfies:

$$R^{⊺}R={RR}^{⊺}=I\text{,}$$ (27)

where I is the identity matrix.

Then, the corresponding Euler angles *α*, *β*, and *γ* can be computed from the rotation matrix R with (Slabaugh 1999):

$$\begin{array}{c} \alpha =atan2\left(\sin\left(\gamma \right)\cos\left(\beta \right),\cos\left(\gamma \right)\cos\left(\beta \right)\right)\\ \beta =atan2\left(-\sin\left(\beta \right),\sqrt{cos^{2}\left(\beta \right)cos^{2}\left(\alpha \right)+cos^{2}\left(\beta \right)sin^{2}\left(\alpha \right)}\right)\\ \gamma =atan2\left(\cos\left(\beta \right)\sin\left(\alpha \right),\cos\left(\beta \right)\cos\left(\alpha \right)\right) \end{array}\text{.}$$ (28)

### Online supplemental material

Fig. S1 describes data composition, Fig. S2 details SpinX pipelines for automated label generation, Fig. S3 evaluates automated label generation using SpinX, Fig. S4 explains data augmentation techniques, Fig. S5 evaluates two SpinX models using IoU metric, Fig. S6 evaluates two SpinX models using Loss of function metric, Fig. S7 are example segmentations from different architectures, Fig. S8 evaluates SpinX Stage 3 for accuracy manually, Fig. S9 on cell cortex eccentricity, Fig. S10 presents spindle length and width measured through SpinX, Fig. S11 outlines analytical solution for 3D Ray-tracing, Fig. S12 compares Refined and Old SpinX algorithms for recording spindle pole positions, Fig. S13 shows increased spindle rotation following CENP-E inhibition, Fig. S14 shows no increase in spindle rotation following MARK2 inhibition, and Fig. S15 presents change in MARK2-YFP localization following its inhibition. Video 1 summarizes SpinX spindle and cortex tracking features. Table S1 shows comparison of SpinX with previous software for spindle and cell cortex detection and tracking. Table S2 shows differences between SpinX-base and SpinX-optimized. Table S3 shows evaluation of SpinX-base and SpinX-optimized models. Table S4 shows evaluation of annotation. Table S5 shows spindle tracking evaluation. Table S6 shows parameters used for PSF simulation.

## Supplementary Material

Table S1 shows comparison of SpinX with previous software for spindle and cell cortex detection and tracking.

Table S2 shows differences between SpinX-base and SpinX-optimized models.

Table S3 shows evaluation of SpinX-base and SpinX-optimized models.

Table S4 shows evaluation of annotation.

Table S5 shows spindle tracking evaluation.

Table S6 shows parameters used for PSF simulation.

## Data Availability

The source code of SpinX can be found at https://github.com/Draviam-lab/spinx_local.
