# Supplementary material for: Deep learning techniques and mathematical modeling allow 3D analysis of mitotic spindle dynamics
Source: J Cell Biol. 2023 Mar 2;222(5):e202111094. doi: 10.1083/jcb.202111094 (PMC9998659; doi:10.1083/jcb.202111094)
Supplement: Table S1 — shows comparison of SpinX with previous software for spindle and cell cortex detection and tracking. [file JCB_202111094_TableS1.docx]

| **Author** | **Software** | **Public** | **Model system** | **Method** | **Contour** | **Time** | **Space** | **Spindle** | **Cortex** | **Auto** | **Condition** | **Year** |
| --- | --- | --- | --- | --- | --- | --- | --- | --- | --- | --- | --- | --- |
| Held et al. | CellCognition | **Y** | Human HeLa | Machine Learning (SVM) | **N** | **Y** | **N** | **Y** | **N** | **Y** | Live cells | 2010 |
| Corrigan et al. | Spindle3D | **N** | Human HeLa | Manual spindle pole selection (brightest  spot) | **N** | **Y** | **Y** | **Y** | **N** | **N** | Live cells | 2013 |
| Decarreau et al. | - | **N** | Human HeLa | Spindle poles as brightest spot | **N** | **N** | **Y** | **Y** | **N** | **N** | Fixed cells | 2014 |
| Larson and Bement | Spindlometer | **N** | Xenopus laevis  embryo | Manual initialization of spindle major axis | **Y** | **Y** | **N** | **Y** | **Y** | **N** | Live cells | 2017 |
| Kushi et al. | MatQuantify | **Y** | Human HeLa | Machine Learning (SVM) | **Y** | **N** | **N** | **Y** | **N** | **Y** | Fixed cells | 2017 |
| Sommer et al. | CellCognition Explorer | **Y** | Human HeLa | Machine Learning (SVM) | **N** | **Y** | **N** | **Y** | **N** | **Y** | Live cells | 2017 |
| Stringer et al. | CellPose | **Y** | Various | Deep Learning (U-Net model) | **Y** | **N** | **N** | **N** | **Y** | **Y** | Fixed cells | 2021 |
| Kletter et al. | Spindle3D | **Y** | Various | Conventional thresholding methods (Otsu) | **Y** | **N** | **Y** | **Y** | **N** | **Y** | Fixed and live cells | 2022 |
|  | | | | | | | | | | | | |
| Dang et al. | SpinX | **Y** | Human HeLa | Deep Learning (Mask R-CNN) | **Y** | **Y** | **Y** | **Y** | **Y** | **Y** | Live cells | 2022 |

**Supplementary Table 1.** Comparison of SpinX with previous software for spindle and cell cortex detection and tracking.
