# Supplementary material for: Deep learning techniques and mathematical modeling allow 3D analysis of mitotic spindle dynamics
Source: J Cell Biol. 2023 Mar 2;222(5):e202111094. doi: 10.1083/jcb.202111094 (PMC9998659; doi:10.1083/jcb.202111094)
Supplement: Table S2 — shows differences between SpinX-base and SpinX-optimized models. [file JCB_202111094_TableS2.docx]

|  | Base model | Optimized model |
| --- | --- | --- |
| Annotation | Less experienced | Refined annotations by experts |
| Sample size | 800 (cell mem.)  900 (spindle) | 1300 (cell mem.)  1390 (spindle) |
| No. Epoch | 200 | 500 |
| Weight decay | 0.0001 | 0.001 |

**Supplementary Table 2.** Differences between SpinX-base and SpinX-optimized. Table shows differences in annotation quality, sample and hyperparameter values between the base and optimized models.
