# Supplementary material for: Deep learning techniques and mathematical modeling allow 3D analysis of mitotic spindle dynamics
Source: J Cell Biol. 2023 Mar 2;222(5):e202111094. doi: 10.1083/jcb.202111094 (PMC9998659; doi:10.1083/jcb.202111094)
Supplement: Table S3 — shows evaluation of SpinX-base and SpinX-optimized models. [file JCB_202111094_TableS3.docx]

|  |  | Training | | | Validation | | | |
| --- | --- | --- | --- | --- | --- | --- | --- | --- |
| Type | Model | Loss | AP | mIoU | | Loss | AP | mIoU |
| Spindle | Base | 0.2293 | 0.976 | 0.874 | | 0.2406 | 0.867 | 0.854 |
|  | Optimized | 0.1928 | 0.988 | 0.861 | | 0.2634 | 0.920 | 0.852 |
| Cell membrane | Base | 0.1467 | 0.975 | 0.682 | | 0.0838 | 0.866 | 0.728 |
|  | Optimized | 0.1761 | 0.989 | 0.758 | | 0.0712 | 0.990 | 0.850 |

**Supplementary Table 3.** Evaluation of SpinX-base and SpinX-optimized models. Table shows evaluation results of base and optimized models through comparisons between Loss, mean AP and mean IoU computed from training and validation datasets.
