# Supplementary material for: Deep learning techniques and mathematical modeling allow 3D analysis of mitotic spindle dynamics
Source: J Cell Biol. 2023 Mar 2;222(5):e202111094. doi: 10.1083/jcb.202111094 (PMC9998659; doi:10.1083/jcb.202111094)
Supplement: Table S4 — shows evaluation of annotation. [file JCB_202111094_TableS4.docx]

|  | Error in training annotation | Error validation annotation |
| --- | --- | --- |
| Spindle | 31% (N=900) | 46% (N=300) |
| Cell membrane | 44% (N=800) | 38% (N=250) |

**Supplementary Table 4.** Evaluation of annotation. Table shows percentage of annotations with errors. These images were identified, re-annotated by an expert, and subsequently used for training and validation of the optimized model.
