# Supplementary material for: Deep learning techniques and mathematical modeling allow 3D analysis of mitotic spindle dynamics
Source: J Cell Biol. 2023 Mar 2;222(5):e202111094. doi: 10.1083/jcb.202111094 (PMC9998659; doi:10.1083/jcb.202111094)
Supplement: Table S5 — shows spindle tracking evaluation. [file JCB_202111094_TableS5.docx]

Corrected

| N | Total time frame | Spindle height | Spindle width | Spindle length |
| --- | --- | --- | --- | --- |
| 10 | 210 | 29%  60 out of210 | 52%  109 out of 210 | 48%  101 out of 210 |

**Supplementary Table 5.** Spindle tracking evaluation. Spindle tracking evaluation of 10 random live-cell movies. The proportion of corrections were analysed for spindle height, width and length axes.
