# Supplementary material for: Deep learning techniques and mathematical modeling allow 3D analysis of mitotic spindle dynamics
Source: J Cell Biol. 2023 Mar 2;222(5):e202111094. doi: 10.1083/jcb.202111094 (PMC9998659; doi:10.1083/jcb.202111094)
Supplement: Table S6 — shows parameters used for PSF simulation. [file JCB_202111094_TableS6.docx]

| **Parameter** | **Description** | **Value** | **Reference** |
| --- | --- | --- | --- |
| NA | NA of the objective lens | 1.4/1.42 | DeltaVision |
| *n_s_* | RI of the sample | 1.34 (Cytosol) | (Hassani & Kreysing 2019) |
| *n_i_* | RI of the immersion | 1.522 | DeltaVision |
| *λ* | Wavelength | FITC: 490nm/525nm | DeltaVision |
|  |  | TRITC: 555nm/605nm | DeltaVision |
|  |  | mCherry: 572nm/632nm | DeltaVision |
|  |  | CY5: 645nm/705nm | DeltaVision |
| M | Magnification | 100/160 | DeltaVision |
| *t* | Working distance | 150 nm | DeltaVision |
| *xd,yd* | Lateral resolution | 0.100 *µ*m | DeltaVision |
| *zd* | Axial resolution | 0.250 *µ*m | DeltaVision |
| X | PSF array width | 256 | - |
| Y | PSF array height | 256 | - |
| Z | PSF array depth | 128 | - |

**Supplementary Table 6.** Parameters used for PSF simulation.
